# *Rorippa islandica*: a genetically accessible dicot model system to study flooding tolerance

**DOI:** 10.1093/plphys/kiag254

**Published:** 2026-04-29

**Authors:** Malte M Bartylla, Emma L R Düthorn, Jana T Müller, Markus Wirtz, Hans van Veen, Rashmi Sasidharan, Angelika Mustroph

**Affiliations:** Department of Plant Physiology, University of Bayreuth, Bayreuth, Germany; Department of Plant Physiology, University of Bayreuth, Bayreuth, Germany; Department of Plant Physiology, University of Bayreuth, Bayreuth, Germany; Centre for Organismal Studies, Heidelberg University, Heidelberg, Germany; Cluster of Excellence GreenRobust, Heidelberg University, Heidelberg, Germany; Evolutionary Plant Ecophysiology, Groningen Institute for Evolutionary Life Sciences, University of Groningen, Groningen, The Netherlands; Plant Stress Resilience, Institute of Environmental Biology, Utrecht University, Utrecht, The Netherlands; Department of Plant Physiology, University of Bayreuth, Bayreuth, Germany

## Abstract

Most crop species cannot survive prolonged flooding events. Within the Cardamineae tribe of the Brassicaceae family, several wild species display high flooding tolerance and are therefore attractive study systems to unravel tolerance mechanisms. However, the genetic recalcitrance of many of these species has prevented detailed mechanistic studies of observed tolerance traits. Here, *Rorippa islandica* was identified as a genetically accessible diploid species with high submergence tolerance. Comparison of its submergence transcriptome with that of another diploid species from the same genus, the submergence-sensitive *R. stylosa*, revealed a strong and partially overlapping transcriptomic response to 48 h submergence. It also revealed *RiBCA3* as a potential tolerance gene contributing to the higher submergence survival of *R. islandica.* Successful CRISPR-Cas9–mediated knockout of *RiBCA3* confirmed the suitability of this species for genetic transformation. However, although it was hypothesized that *RiBCA3* might have an important function in carbon fixation under water, no differences in submergence survival or underwater photosynthesis were observed between wild-type and *bca3* knockout lines. The molecular mechanisms of submergence tolerance of *Rorippa islandica* are therefore not yet understood. This work demonstrates the suitability of *Rorippa islandica* for molecular genetics studies and presents a promising dicot model for investigation of underlying tolerance mechanisms, especially for comparative studies with Arabidopsis. This species might contribute to knowledge on flood tolerance in dicots, particularly *Brassica* oilseed crops and vegetables, while current knowledge is based primarily on rice (a monocot) studies.

## Teaser

*Rorippa islandica* is presented here as a flooding-tolerant Brassicaceae species whose genetic accessibility might help elucidate mechanisms underlying flooding tolerance.

## Introduction

Ecological niches in terms of water availability range from dry conditions (deserts and savannah), normal water availability to soils with high water content (swamps, shores) (Silvertown et al. 2015) up to aquatic habitats (rivers, lakes) (Koga et al. 2024). Plant populations have adapted to the demands of each of these specific environmental niches, but only a few plant species have adapted to water-rich environments. This is due to the multiple problems arising from low gas diffusion rates in water compared with air, resulting in a shortage of oxygen and carbon dioxide (CO_2_) within plant tissues, and an accumulation of the gaseous hormone ethylene. Several adaptive strategies have evolved to either avoid low gas diffusion rates through morphological and anatomical modifications (low-oxygen escape syndrome) or cope with low gas diffusion (low-oxygen quiescence syndrome) (Voesenek and Bailey-Serres 2009; Lin et al. 2024). Within the plant kingdom, there is large variation in the type and extent of these adaptations.

The low-oxygen escape syndrome can have many facets—for example, aerenchyma formation with different mechanisms of formation (Takahashi et al. 2014), elongation of aerial organs to reach the water surface, formation of adventitious or specialized roots, and the formation of barriers in roots to avoid radial oxygen loss (Voesenek and Bailey-Serres 2015). In conditions of long or permanent submersion, plants have evolved other adaptive mechanisms to improve gas uptake from the surrounding water—for example, thinner leaf cuticle, lower number of stomata, and, in some cases, more dissected leaves (Koga et al. 2024).

The model species *Arabidopsis thaliana* has been used extensively for research on flood tolerance. However, it is not adapted to grow in water-rich environments, neither partially (ie, waterlogging) nor fully (ie, submergence). Within 2 to 4 weeks, Arabidopsis plants die under either stress condition, depending on other factors like temperature and light availability (Vashisht et al. 2011; van Veen et al. 2016; Meng et al. 2020; Ventura et al. 2020; van Veen et al. 2024). Despite not being a true flood-adaptive species, Arabidopsis has been the model of choice due to its genetic tools and ease of transformation. The only other model system with higher flooding tolerance is rice, which is a monocot. The scientific community lacks a dicot model to understand the molecular basis of flood-adaptive traits and flood tolerance. An ideal candidate would be a very flood-tolerant species closely related to Arabidopsis, which provides an opportunity to leverage all the knowledge and experience obtained from Arabidopsis.

In previous studies, we and others have identified relatives of Arabidopsis that are adapted to flooding conditions, all belonging to the Cardamineae tribe (Akman et al. 2012; van Veen et al. 2014; Sasidharan et al. 2013; Shimizu-Inatsugi et al. 2017; Hwang et al. 2020; Sun et al. 2020; Müller et al. 2021; van Veen et al. 2024). Even within this relatively narrow group of plants, which all belong to the tribe Cardamineae, several adaptive survival strategies have been observed, based on the habitat in which these plant species occur. For example, *Nasturtium officinale* grows in running water along rivers and uses the low-oxygen escape syndrome (Müller et al. 2021), while some *Rorippa* species rather occupy habitats with standing water and use the low-oxygen quiescence syndrome (van Veen et al. 2024).

In our previous phylotranscriptomic approach comparing 5 Brassicaceae species, ranging from the sensitive species, *Arabidopsis thaliana*, to the most tolerant species, *Rorippa sylvestris*, we identified a large number of differentially expressed genes (DEGs) after 24 and 48 h of submergence for each species, together with a list of differentially expressed orthogroups (OGs). Many OGs showed similar expression patterns across species (eg, genes coding for enzymes involved in the carbon starvation response) (van Veen et al. 2024). However, this starvation response was attenuated in tolerant plant species, hinting at more efficient photosynthesis in submerged leaves. Interestingly, most genes associated with photosynthesis were not differentially expressed under submergence. An exception was the gene *Beta Carbonic Anhydrase 3* (*BCA3*), a highly induced gene in the tolerant species *Rorippa sylvestris* (van Veen et al. 2024). Carbonic anhydrases (CAs) catalyze the interconversion between CO_2_ and HCO_3_^−^ (bicarbonate), and plants contain several isoforms in 3 groups: α-CAs, β-CAs, and γ-CAs (DiMario et al. 2017).

Bicarbonate is the dominant inorganic carbon form in water, but it can only be taken up and used for photosynthesis in some aquatic plant species (Maberly and Spence 1983; Pedersen et al. 2013; Maberly and Gontero 2017; Iversen et al. 2019; Huang et al. 2020), and therefore BCAs might hold relevance for underwater photosynthesis in *R. sylvestris*.

We therefore hypothesized that BCA3 could be one component contributing to the submergence tolerance of *Rorippa sylvestris* and related Brassicaceae species. However, the most tolerant species studied in our work are polyploid and self-incompatible (*Cardamine pratensis*, *R. sylvestris*, *R. amphibia*) (van Veen et al. 2024). Both traits complicate genetic studies with those species.

Here we sought to test whether we could find a genetically accessible “model” dicot species from this phylogenetic tribe. In this work, we selected two additional diploid *Rorippa* species that have not been studied at the molecular level yet, *R. islandica* and *R. stylosa* (synonym *R. pyrenaica*). *R. islandica* was selected since it represents the first *Rorippa* species with a sequenced genome (Brassicales Map Alignment Project, Department of Energy's Joint Genome Institute; http://bmap.jgi.doe.gov/) and can reproduce by autogamy. We selected *R. stylosa* (= *R. pyrenaica*) as a potentially flooding-sensitive *Rorippa* species, based on the Ellenberg numbers for moisture and its growth habitat (Ellenberg and Leuschner 2010) (Table 1).

**Table 1:** Ellenberg moisture values and characteristics of the studied species.

Species	Ellenberg moisture value (Ellenberg and Leuschner 2010)	Ploidy and subgenomes (Han et al. 2024)	Fertilization	Lifestyle
*Arabidopsis thaliana*	4	diploid	Selfing	Annual
*Rorippa amphibia*	10	tetraploid (BBBB)	Self-incompatible	Perennial
*Rorippa austriaca*	7=	diploid (AA)	Self-incompatible	Perennial
*Rorippa islandica*	8=	diploid (DD)	Selfing	Annual
*Rorippa palustris*	8=	tetraploid (BBDD)	Selfing	Annual
*Rorippa stylosa (pyrenaica)*	5	diploid (DD)	Self-incompatible	Perennial
*Rorippa sylvestris*	8=	tetraploid (AAAA)	Self-incompatible	Perennial

First, we determined the global transcriptome profiles of both species under submergence by RNA sequencing (RNA-seq), similar to our previous work. Then, we established a transformation protocol for *R. islandica* and used this to study the proposed tolerance gene, *RiBCA3*, through the creation of *RiBCA3* knockout plants. While we did not see any obvious phenotypes, our experiments demonstrate that this species can be a model for further analyses of potential candidate tolerance genes. Therefore, this study provides a basis for future studies to decipher the molecular mechanisms of flooding tolerance and identifies a dicot, diploid flood-tolerant model species for future studies.

## Materials and methods

### Plant material and growth conditions

Seeds of *Rorippa islandica*, obtained from Brassibase (Koch et al. 2012), *Rorippa palustris* (https://wildblumen.at), and *A. thaliana* ecotype Col-0 were propagated generatively in-house. The dry seeds were sterilized in a chlorine gas atmosphere for 45 min and germinated on solid MS medium (1×MS-salts, 1% [w/v] sucrose, 1% [w/v] agar, pH 5.7) in growth cabinets under long-day conditions (22 °C; 16 h of light/8 h of darkness; 100 µmol photons m^−2^ s^−1^). Only *A. thaliana* was stratified. Seedling assays were done by transferring the seedlings either to MS medium for *A. thaliana* or to Hoagland medium [280 µM Ca(NO_3_)_2_, 100 µM NH_4_H_2_PO_4_, 200 µM MgSO_4_, 600 µM KNO_3_, 5 µM Fe-HBED, 1% (w/v) sucrose, 5 mM 2-(N-morpholino)ethanesulfonic acid, 1% (w/v) type E agar, pH 5.7] for *R. islandica* 7 days after sowing. *Rorippa sylvestris* and *Rorippa amphibia* originate from plants used in Stift et al. (2008) and Akman et al. (2012). *Rorippa austriaca* was kindly provided by Mirka Macel (University Tübingen, Germany), and *Rorippa stylosa* (synonym *R. pyrenaica*) was obtained from the Botanical Garden Bern (Switzerland). The latter 4 were vegetatively propagated as described earlier (Akman et al. 2012). *A. thaliana* seedlings were pricked in a soil mixture consisting of sowing and potting soil (Ökohum GmbH, Germany), Einheitserde CL ED73 (Einheitserdewerke Werkverband e.V., Germany), and Vermiculit G (Dämmstoff-Fabrik Klein GmbH, Germany) in a ratio of 3:3:1, which was mixed 2:1 with sand for submergence experiments. *Rorippa* seedlings or root sprouts were pricked in a nutrient solution-saturated soil mixture, described in Benschop et al. (2005). Soil used in long-term submergence experiments was steamed at temperatures between 80 °C and 90 °C for at least 2 h. All species were further cultivated in growth chambers under short-day conditions (22 °C; 8 h of light/16 h of darkness; 100 µmol photons m^−2^ s^−1^), where the experiments also took place. Plants referred to as adult plants were grown for 3 weeks on soil after pricking.

### Stress treatments

Stress treatments started 2 h after the onset of light. Submergence experiments were conducted in transparent plastic boxes, filled with tap water the day before. Control plants were kept in empty boxes. For the study of pH-dependent transcriptional regulation, 0.5 mg/L 2-(N-morpholino)ethanesulfonic acid was added to the water, and pH values were adjusted using HCl or NaOH. The inhibition of ethylene receptors was achieved by the addition of 10 µM silver thiosulfate to the water, freshly prepared from silver nitrate and sodium thiosulfate in a ratio of 1:3. Ethylene treatments were performed in airtight desiccators with the use of ethephon according to Tucker et al. (2017) (final concentration 10 ppm ethylene). Hypoxia stress was induced by gassing the plants in airtight desiccators either with nitrogen or ambient air as a control. From adult plants, young healthy leaves were sampled, while from seedlings, rosettes and roots were sampled separately, and plant material was frozen in liquid nitrogen directly after harvesting. Survival assays were carried out like all other submergence experiments. At every time point, 8 plants were taken randomly for each species and were able to recover for 2 weeks under short-day conditions with normal irrigation. After that, the ability to form new leaves was used as an indicator for the survival of the plants.

### Phenotyping for growth deficits

Whole rosettes of 5 adult plants per genotype were cut off. Weight was determined right after harvest and after drying for 3 days at 60 °C. Genotypes were evenly distributed on plates and the length of the primary root was measured 7 or 10 days after the transfer from germination plates for *A. thaliana* and *R. islandica*, respectively.

### PCR analysis

RNA extraction, complementary DNA (cDNA) synthesis, and reverse transcription quantitative PCR analysis were carried out as described in Müller et al. (2021). Deviating from this, TRIzol (Thermo Fisher Scientific Inc., USA) was used for some samples. As reference genes, *SERINE/THREONINE PROTEIN PHOSPHATASE 2A* (*PP2A*, AT1G69960) and *UBIQUITIN CONJUGATING ENZYME 9* (*UBC9*, AT4G27960) for *A. thaliana* and orthologous genes of *ACTIN 2* (*ACT2*, AT3G18780) and *BETA-6 TUBULIN* (*TUB6*, AT5G12250) for *Rorippa* species were used together. Reverse transcription PCR with cDNA was performed using Taq polymerase and subsequent agarose gel analysis. Samples of 2 separate adult plants or several seedlings were pooled for each measurement. Specific primers used are listed in Table S2.

### RNA sequencing and bioinformatic analysis

For RNA-seq, total RNA was extracted from frozen, ground leaf material using ISOLATE II RNA Plant Kit (Meridian Bioscience Inc., USA) following the manufacturer's instructions. Quality control, library preparation with a selection for polyadenylation, and sequencing on an Illumina NovaSeq System in 150 bp paired-end mode were performed by Genewiz GmbH (Germany). Raw sequencing data were deposited in NCBI's Gene Expression Omnibus and are accessible through GEO Series accession number GSE295090.

RNA-seq raw data were checked by FastQC (v0.12.0; Andrews 2010) and aligned against the *R. islandica* transcriptome (Assembly Version v1, Annotation Version v1.1; Brassicales Map Alignment Project, DOE-JGI; https://bmap.jgi.doe.gov/) for both species using kallisto (v0.46.1) (Bray et al. 2016). Mapping statistics are presented in Table S1. Subsequently, isoforms of the same gene were summed up according to the *R. islandica* gene identifiers. Differential gene expression analysis was performed twice (lfcThreshold = 0 and lfcThreshold = 1 for all genes and differentially expressed genes, respectively) with the R-package DESeq2 (v.1.34.0; Love et al. 2014) and further filtered on an adjusted *P* < 0.05. DESeq2 was used for PCA as well.

To compare gene expression data to our previous analysis, the mapped reads were also assigned to OGs previously identified (van Veen et al. 2024) and further processed to obtain differentially expressed OGs, exactly as described elsewhere (van Veen et al. 2024).

For GO analysis, AGI codes already annotated in the *R. islandica* transcriptome were extracted, and duplicates were removed. A statistical overrepresentation test (Mi et al. 2019) with the GO biological process complete (Carbon and Mungall 2023) was carried out in PANTHER (v18.0; Thomas et al. 2022), separately for induced and repressed genes. As a test type, Fisher's exact test and a correction for the false discovery rate were used against all AGI codes detected at least once in the respective species.

The difference between *R. stylosa* and *R. islandica* coding sequences was also explored in addition to differential messenger RNA abundance. Reads were mapped to the reference transcriptome of *R. islandica* with the Burrows–Wheeler Aligner maximal exact matches algorithm (Li and Durbin 2010). The resulting alignments were arranged in a variant call format and pileup formats. These served as a basis for coverage estimation. An average depth higher than 20 for a gene was considered sufficient to be included in the mutation analysis. Variants (single-nucleotide polymorphisms and insertions/deletions) were called with bcftools (Li 2011). Variants with a Phred score greater than 40 were considered reliable. The extent to which nucleotide variation resulted in amino acid sequence variants was assessed by reconstituting the variant with 50 upstream and 50 downstream nucleotide sequences and a BLASTX search against the *R. islandica* proteome (Altschul et al. 1990).

### Generation of plasmids

Overexpression plasmids were generated by Gateway cloning. Inserts of the coding sequence of *BCA3* were amplified from cDNA, simultaneously adding attB-sites. BP reaction was performed in *pDONR221* (Thermo Fisher Scientific Inc., USA) and LR reaction in *pMDC43-HA* or *pMDC43-GFP* (Curtis and Grossniklaus 2003), both according to the manufacturer's specifications (Gateway BP and LR Clonase II, Thermo Fisher Scientific Inc., USA). For CRISPR-Cas9 knockouts of *BCA3*, *pHSE401* (Xing et al. 2014) was used and modified to its target sequence as described by the authors. The targets were picked based on the predicted activity and target specificity of the Cas9 enzyme (Hsu et al. 2013b; Doench et al. 2014), by use of the software Geneious Prime 2023.2.1 (https://www.geneious.com). For establishment of *R. islandica* transformation, the vector pMDC32-GFP (Kuriyama et al. 2019) was used.

For yeast transformation, insert fragments were amplified and cloned into *pDD506* according to Rai et al. (2022), kindly provided by James V. Moroney from Louisiana State University, together with a *pDD506-NCE103* positive control. Either cDNA from plants or *pMDC43-HA* plasmids carrying the *BCA3* coding sequence were used for insert generation. Phusion High-Fidelity DNA-Polymerase (Thermo Fisher Scientific Inc., USA) was used to amplify all insert fragments. For cloning procedures, competent *Escherichia coli* DH10b cells were used and transformed by heat shock. Selection was done on lysogeny broth medium (5 g/L yeast extract, 10 g/L tryptone, 10 g/L NaCl, 1.5% [w/v] agar, pH 7.0) supplemented with either 50 mg/L kanamycin or 50 mg/L carbenicillin. Plasmids of positive transformants were confirmed by Sanger sequencing and restriction digestion. Specific primers used are listed in Table S2.

### Transformation of *Rorippa islandica* and *Arabidopsis thaliana*

Plasmids were transformed into *Agrobacterium tumefaciens* strain GV3101::pMP90K (Koncz and Schell 1986) using the freeze–thaw method (Chen et al. 1994). *A. thaliana* was transformed by floral dip (Clough and Bent 1998) with 10% (w/v) sucrose and 0.02% (v/v) Silwet L-77 added to the *Agrobacterium* solution. For *R. islandica* this procedure was further modified by pelleting the *Agrobacterium* solution at an OD_600_ of 0.7 to 0.8 for 20 min at 5,000 rpm and 4 °C, subsequently resuspending them by shaking at 200 rpm and 30 °C in the same volume of infiltration medium (50 g/L sucrose, 10 mM MgCl_2_, 0.1 mg/L 6-benzylaminopurine). Just before dipping, 0.03% (v/v) Silwet L-77 was added. Seeds of both species were selected for positive transformants on MS medium containing either 50 mg/L kanamycin or 20 mg/L hygromycin. Candidates were screened by genomic DNA extraction followed by PCR with subsequent agarose gel analysis for the presence of the insert or Sanger sequencing of the CRISPR-Cas9 target sequence. Plants of the T3 generation with a homozygous knockout and the loss of the inserted Cas9 enzyme, or with a homozygous insertion of the overexpression construct were used for further experiments, respectively.

### Microscopy

Seedlings of *R. islandica* (transformed with pMDC32-GFP, T2 generation) were checked for a green fluorescent protein (GFP) signal using an optical microscope (Leica MZ10F) with an external light source (EL6000) and a filter (excitation 460–500 nm, dichroic mirror 505 nm, emission 512–542 nm), all obtained from Leica Microsystems GmbH, Germany.

Roots of adult plants of *R. islandica* and *R. sylvestris* were harvested and subsequently used to make cross sections with a handheld razor blade. Cross sections were photographed using a microscope (Leica DM2500 LED, Leica Microsystems GmbH, Germany).

### Protein extraction and western blot

For protein extraction, 50 mg of 7-day-old seedlings were ground in liquid nitrogen, thawed in 50 µL urea buffer (5% [w/v] sodium dodecyl sulfate [SDS], 16.5% [v/v] glycerine, 4 M urea, 0.2 g/L bromophenol blue), and incubated at 95 °C for 10 min. After a centrifugation of 15 min at 4 °C and 16,100 × *g*, samples were loaded onto a polyacrylamide gel (stacking gel: 3% [v/v] N, N′-Methylenebisacrylamide, 0.125 M Tris-HCl pH 6.8; resolving gel: 10% [v/v] N, N′-Methylenebisacrylamide, 375 mM Tris-HCl pH 8.8; both gels 0.1% [w/v] SDS, 0.0005% [w/v] ammonium persulfate, 0.001% TEMED) against a PageRuler Plus prestained protein ladder (#26619, Thermo Fisher Scientific Inc., USA). Gel electrophoresis was performed in a buffer consisting of 25 mM Tris, 192 mM glycine, and 0.1% [v/v] SDS and subsequently blotted onto a methanol-activated ROTI polyvinylidene fluoride 0.45 transfer membrane (Carl Roth GmbH + Co. KG, Germany) in a buffer of the same composition but with 20% [v/v] methanol instead of the SDS. The membrane was incubated in 2.5% skimmed milk powder, dissolved in 1 × PBS-T (137 mM NaCl, 2.7 mM KCl, 10 mM Na_2_HPO_4_, 1.8 mM KH_2_PO_4_, 0.1% [v/v] Tween-20, pH 7.4), overnight. The protein of interest was detected using an anti-HA antibody (H9658, Merck KGaA, Germany; 1:3,000 diluted in the skimmed milk solution, as previously described), followed by an anti-mouse horseradish peroxidase antibody (#4759, Carl Roth GmbH + Co. KG, Germany; 1:10,000 diluted as before), each incubated for 1 h. Chemiluminescence was triggered using Amersham ECL Prime Western Blotting Detection Reagent (Global Life Sciences Solutions USA LLC, USA) and detected by a low-light imaging system. Total protein was stained with 0.1% (w/v) Coomassie brilliant blue R-250 in 10% (v/v) acetic acid and 50% (v/v) methanol, which was destained with a solution made of 7% (v/v) acetic acid and 20% methanol (v/v) before imaging. The membrane was washed several times using 1 × PBS-T between each step.

### Underwater photosynthesis

The underwater photosynthesis was indirectly measured by the oxygen production using a PyroScience system (FireSting-O_2_ FSO2-C4, temperature sensor TDIP15, contactless oxygen sensor vial OXVIAL4 with adapter ring ADVIAL4, fiber optic cable SPFIB BARE; PyroScience GmbH, Germany) and the Pyro Oxygen Logger (v3.31.9) software. Water gassed with air was used to calibrate the sensor to 100% oxygen saturation. A solution of 2 mM NaHCO_3_ and 0.1 mM phosphate buffer of either pH 6.3 or pH 8.3 was added to the sensor vial, constantly stirred at 500 rpm. The oxygen content was measured in ppm and corrected for temperature every second. A single leaf of the plant to be examined was added and used for measurements at both pHs. The sensor vial was illuminated with light of 100 µmol photons m^−2^ s^−1^, the oxygen content was measured for 15 min, and the oxygen production was calculated by plotting the oxygen values of the last 10 min against the time. Control measurements in the dark and without leaves were carried out as well. Values were normalized by the dry weight of the measured leaf.

### Extraction and analysis of sugars

Leaf material of 2 plants was combined in 1 sample, and sugar and starch were measured according to Ambros et al. (2022). Extraction volumes were adjusted to the respective fresh weights.

### Extraction and analysis of amino acids

Leaf material was ground in liquid nitrogen and vortexed in 250 µL of 0.1 M HCl and 4% polyvinylpyrrolidone for 15 min at 4 °C. After centrifugation for 5 min at 14,000 rpm, amino acids were extracted from the supernatant using the AccQ-Tag Ultra Derivatization Kit (Waters Corporation, USA) according to the manufacturer's specifications. Amino acids were measured using reverse-phase high-performance liquid chromatography as described in Wirtz et al. (2004). Samples from 2 separate plants were used as technical replicates for each measurement.

### Extraction and analysis of fatty acids

Fatty acids from leaf material were extracted and measured using gas chromatography according to Wewer et al. (2013). Samples from 2 separate plants were used as technical replicates for each measurement.

### Yeast transformation

*S. cerevisiae* strain W303-1A-*ΔNCE103* (Rai et al. 2022), kindly provided by James V. Moroney from Louisiana State University, was used. Cells were grown at 30 °C and 5% CO_2_ on YNB_-Trp_ plates, consisting of 1.7 g/L YNB without amino acids and (NH_4_)_2_SO_4_ (Formedium Ltd., UK), 5 g/L (NH4)_2_SO_4_, 20 g/L glucose, 0.6 g/L Drop Out Mix (CSM-HIS-LEU-TRP-URA, MP Biomedicals LLC, USA), 20 g/L Difco Agar (Becton, Dickinson and Company, USA), and 30 mg/L each leucine, uracil, and histidine. The transformation was done according to Chen et al. (1992). In contrast to the protocol, a single colony was picked directly from the plate, and the buffer consisted of 40% (w/v) PEG3350, 100 mM LiAc*2 H_2_O, 10 mM Tris-HCl pH 7.5 and 1 mM EDTA. Subsequent incubation was prolonged to 4 h at 42 °C. Herring sperm (Promega GmbH, Germany) was used as the single-stranded carrier DNA. Positive clones were identified by selection on YNB_Trp, His_ and colony PCR.

### Drop dilution assay

The assay is based on the publication of Rai et al. (2022). YNB_-Trp, -His_ was inoculated with *ΔNCE103* cells bearing different pDD506 plasmids. The cells were incubated at 30 °C, 5% CO_2_, and 220 rpm overnight. The next day, a 1:10 dilution series was prepared starting at an OD_600_ of 0.5, and droplets of it were placed on YNB_-Trp, -His_ Petri dishes. The Petri dishes were prepared in duplicates and were incubated at 30 °C, either adding 5% CO_2_ or using ambient air for 42 h.

### Programs and statistical analyses

Bioinformatic analyses with R (v4.3.1; R Core Team 2022) were carried out in RStudio (Posit Team 2023). Figures were created with GraphPad Prism 9 (GraphPad Software LLC, USA). Venn diagrams were generated with the R-package ggvenn (Yan 2023), and the ridgeline plot was created with the R-package ggridges (Wilke 2024).

Student *t*-tests and One-way analysis of variance (ANOVA) with Tukey's honestly significant difference were calculated with Prism 9. Two-way ANOVA with Tukey's honestly significant difference was performed in R using the R-package multcompView (Graves et al. 2023).

### Accession numbers

Sequence data from this article have been deposited in NCBI's Gene Expression Omnibus (GEO accession GSE295090). Gene IDs used in this article are presented in Dataset S1.

## Results

### Characterization of the submergence response of two *Rorippa* species

**Figure 1:**
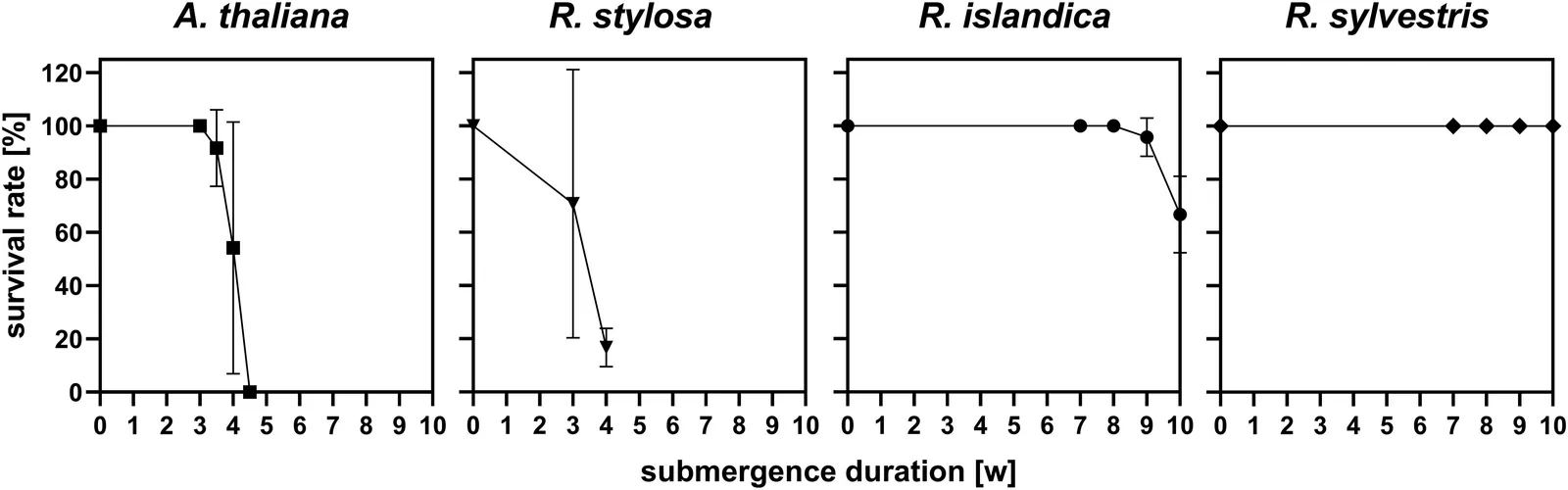
Survival of submergence in day–night rhythm of *A. thaliana* and *Rorippa* species. Plants were subjected to submergence for 3 to 10 weeks and subsequently recovered under normal growth conditions for 2 weeks. Survival was defined as the ability to produce new leaves. Data are means ± SD from 3 independent experiments with 8 plants per replicate and time point.

In our previous work, we showed that *Rorippa sylvestris* and *R. palustris*, 2 tetraploid species, can survive submergence in the light for up to 10 weeks (van Veen et al. 2024). A similar survival assay was performed with the 2 diploid species *R. islandica* and *R. stylosa*. While about 70% of individuals of *R. islandica* survived 10 weeks of submergence, the tolerance of *R. stylosa* was much lower, ranging between 3 and 4 weeks (Fig. 1), comparable to the sensitive Arabidopsis (Vashisht et al. 2011). The contrasting tolerance of those 2 *Rorippa* species encouraged us to analyze the molecular response of both species to submergence, as an addition to our previous dataset (van Veen et al. 2024).

Plants were grown for 3 to 4 weeks on soil and subsequently treated with submergence under a normal day–night rhythm. The transcriptome changes after 24 and 48 h of stress were analyzed. For easier comparison, the reads from both species were mapped to the sequenced genome of *R. islandica* (BMAP). Mapping statistics were better when compared with a self-assembled transcriptome of *R. stylosa* (Table S1).

**Figure 2:**
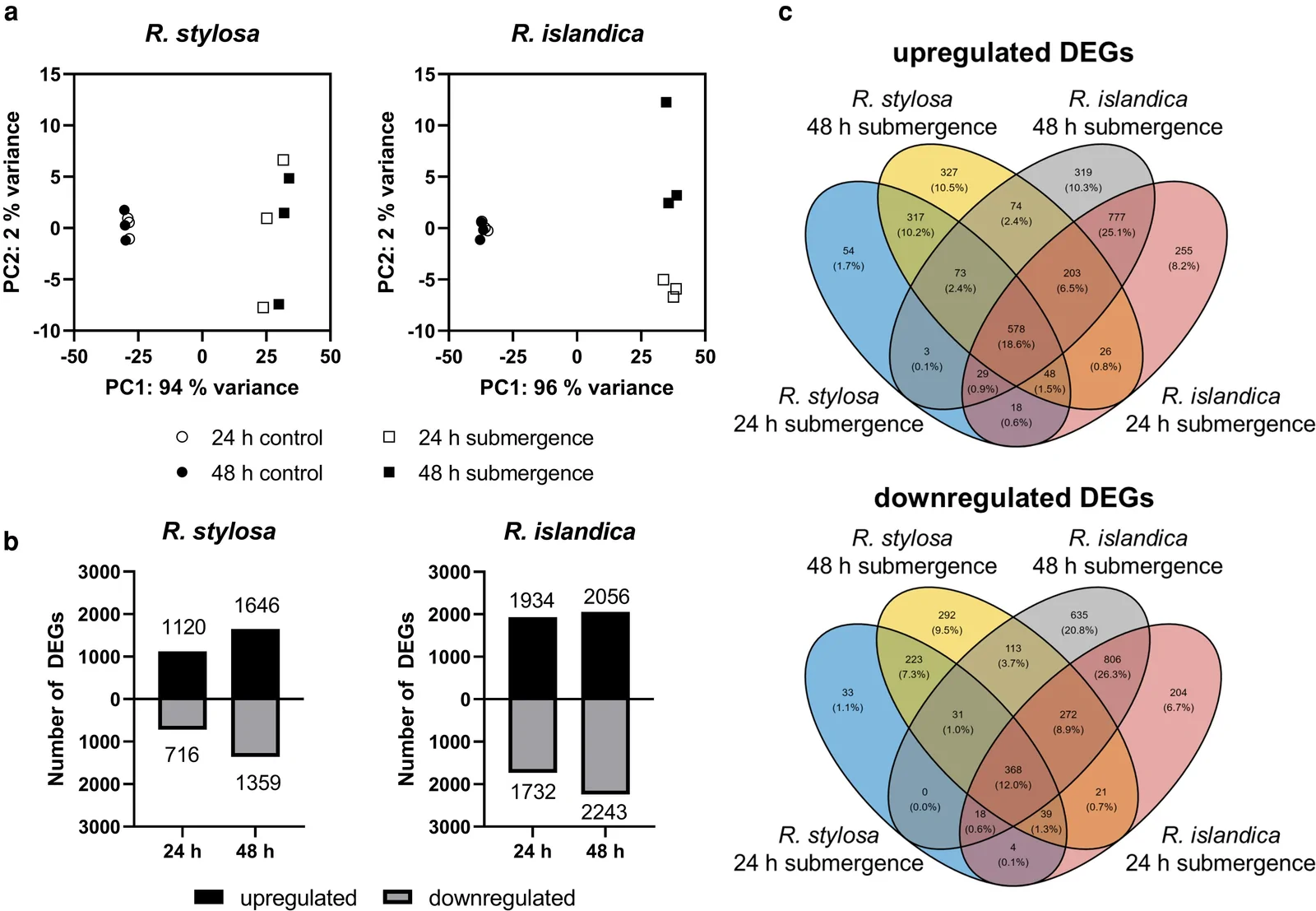
Gene expression is modified after submergence in *R. stylosa* and *R. islandica*. Plants were treated with submergence in a day-night rhythm for 24 and 48 hours, and subsequently, young leaves were harvested for RNA extraction and RNA-seq. (a) PCA of transcript levels of control and stress samples in *R. stylosa* and *R. islandica*. (b) Number of DEGs (adjusted *P* < 0.05 and |log_2_FC| >1) in *R. stylosa* and *R. islandica*. (c) Overlap of DEGs between the 2 species and the 2 time points.

Both plant species exhibited a strong transcriptional response to the stress, and samples were clearly separated by stress treatment (Fig. 2a). Interestingly, *R. stylosa* samples did not show differences between harvest time points, and there was some variation between biological replicates. This could be because they were propagated vegetatively, independently for each replicate, and the growth of the plantlets was not as homogeneous as seedlings from *R. islandica*. *R. islandica* samples separated by stress time point in the second PCA component. The number of DEGs (|log_2_FC| >1 and adjusted *P* < 0.05) in tolerant *R. islandica* was higher compared with sensitive *R. stylosa* per time point and for both up- and downregulated transcripts (Dataset S1, Fig. 2b). Still, there was a large overlap between both species, 953 transcripts, of which 578 were commonly upregulated and 368 were commonly downregulated, the remaining 7 sequences were oppositely regulated between species (Fig. 2c; Dataset S1). This suggests that major transcriptional responses were similar in sensitive and tolerant species, and those might be associated with general tolerance mechanisms of plants, but not with higher tolerance of *R. islandica*.

Gene Ontology (GO) term enrichment analysis of DEGs revealed commonly upregulated processes related to “indole glucosinolate metabolic process”, “defense”, “external stimulus”, “response to hypoxia”, and “response to ethylene” (Dataset S1). Although several GO terms related to hypoxia were enriched, only a part of the hypoxia core response genes were induced during submergence (Figure S1), as described in our earlier studies (Wittig et al. 2021; van Veen et al. 2024). Furthermore, a clear starvation response was observed (Figure S2), as in our previous datasets, despite harvesting samples under illumination. In addition, we speculated that ethylene might be a signal in submerged plants. Indeed, ethylene-responsive genes from *Arabidopsis* were among the submergence-induced genes here (Figure S3). However, there was also some variation between species. In *R. stylosa*, GO terms related to “phytoalexin biosynthesis” were enriched among upregulated genes, while the GO term “cellular response to sucrose starvation” was enriched only in *R. islandica*.

Commonly downregulated genes were enriched in functions related to biosynthetic processes, for example “glucosinolate biosynthesis”, “cell wall modification”, and “carbohydrate derivative biosynthesis” (Dataset S1). Also here, several enriched GO terms were different between species. In *R. islandica*, downregulated genes were enriched in the functions “cell cycle” and “stomata development”. This trend was also visible when looking at specific genes that have been defined as stomata-associated (Figure S4) (Pillitteri and Dong 2013; Chowdhury et al. 2021). In *R. stylosa*, the enriched GO terms were associated with “indoleacetic acid biosynthesis” and “serine family amino acid biosynthesis”.

Since we had speculated that CAs, especially the highly induced *RsBCA3* (van Veen et al. 2024), could contribute to submergence tolerance, we also checked the expression of the genes of all 3 CA families (Figure S5). γ-CAs were not differentially expressed, and among α-CAs, expression of the major isoform *ACA1* was significantly reduced under submergence in both species. Overall expression levels of all other *ACAs* were very low, compared with *ACA1* (Dataset S1). Among β-CAs, both species induced mitochondrial *BCA6*. In addition, plastidic *BCA1* was downregulated, while cytosolic *BCA3* was upregulated more strongly in *R. islandica*. Furthermore, alternative carbon-concentration mechanisms, including C4- and CAM-like photosynthesis, have been described for some plant leaves under water (eg, Keeley 1990; Klavsen et al. 2011; Keeley 2014; Yin et al. 2017; Maberly and Gontero 2017; Iversen et al. 2019), but genes associated with those processes were either not differentially expressed or downregulated (Figure S6) (Goolsby et al. 2018).

### Overlapping response between species

To compare the transcriptomic response of the 2 diploid species to our previous dataset, we used the previously defined OGs that already contained *R. islandica* sequences (van Veen et al. 2024). For this comparison, the data of the 2 newly selected species were analyzed exactly as the previous ones, and expression data of the universal OGs were combined (Dataset S2). Again, a set of 285 OGs was commonly upregulated, and a set of 170 OGs was commonly downregulated across sensitive and tolerant species. The commonly upregulated OGs included starvation-responsive genes such as *MIOX2* and *BCAT-2*, as well as GO terms associated with “response to hypoxia” and “response to ethylene”, as well as defense-related processes (Dataset S2). Commonly downregulated genes were several histones, *beta-amylase 5*, and GO terms related to cell cycle, cell wall, and different biosynthetic processes. Based on these expression patterns, we suggest that those genes are not responsible for the enhanced tolerance of *R. islandica* in comparison to *R. stylosa*.

In addition, there were different OGs induced in different plant species. However, no clear set of genes could be identified with induced expression in 4 tolerant species (*C. pratensis*, *R. palustris*, *R. islandica*, *R. sylvestris*), and no induction in sensitive species. This observation suggests that tolerant species might exhibit different tolerance mechanisms, and species must be compared separately.

### Comparison of the two *Rorippa* species with contrasting submergence response

The good mapping results of *R. stylosa* transcripts to the *R. islandica* transcriptome enabled direct comparison of a sensitive and a tolerant species. There was a massive difference in gene expression between the 2 species under both control and submerged conditions (Dataset S3). For example, *R. islandica* expressed 3,459 transcripts more than *R. stylosa* at the 24-h control time point, while 2,643 transcripts were more highly expressed in *R. stylosa* (Figure S7). About 45% and 35%, respectively, were commonly differentially expressed between species, independent of the treatment (Figure S7). A GO term enrichment analysis revealed mainly differences in functional categories related to defense and immunity in *R. islandica* and toxin catabolism in *R. stylosa* (Dataset S3).

However, very few GO terms were enriched in either species only under stress conditions, namely, glucosinolate biosynthesis, which was more expressed in *R. stylosa*. Therefore, no obvious processes could be associated with the higher submergence tolerance of *R. islandica* compared with *R. stylosa*. But we identified at least 196 genes with significantly higher expression in *R. islandica* compared to *R. stylosa* under submergence, which are also significantly induced by submergence in *R. islandica* (Dataset S4), as candidates to be responsible for enhanced tolerance.

In addition, tolerance could also be associated with sequence variations rather than gene expression changes. To assess this, the raw sequencing reads were used for variant calling, compared to the published *R. islandica* genome. Not surprisingly, for 2 separate species, about 95% of transcripts had sequence variations between *R. stylosa* and *R. islandica* (Figure S8, Dataset S5). Of the sequence variants observed in *R. stylosa*, 38% would encode an alternate peptide sequence compared to the *R. islandica* reference. Fifty percent of genes had fewer than 9 variants of these protein sequence-altering variations (single-nucleotide polymorphisms and insertions/deletions), and 90% of all genes had fewer than 34 variants. These values indicate relatively small genetic differences between the 2 species compared with the relatively large transcriptomic differences.

### Establishment of *R. islandica* as a genetically accessible model system

The analysis of submergence tolerance as well as transcriptome responses to the stress revealed that *R. islandica* could be a good dicot model for flooding studies. It has a high submergence tolerance, compared with the other diploid species *A. thaliana*, *C. hirsuta*, and *R. stylosa*. In addition, it is annual, selfing, has a short generation time, and does not require vernalization for flowering. In contrast, the other diploid *Rorippa* species, *R. stylosa*, is perennial, requires about 16 weeks of vernalization, and is self-incompatible.

**Figure 3:**
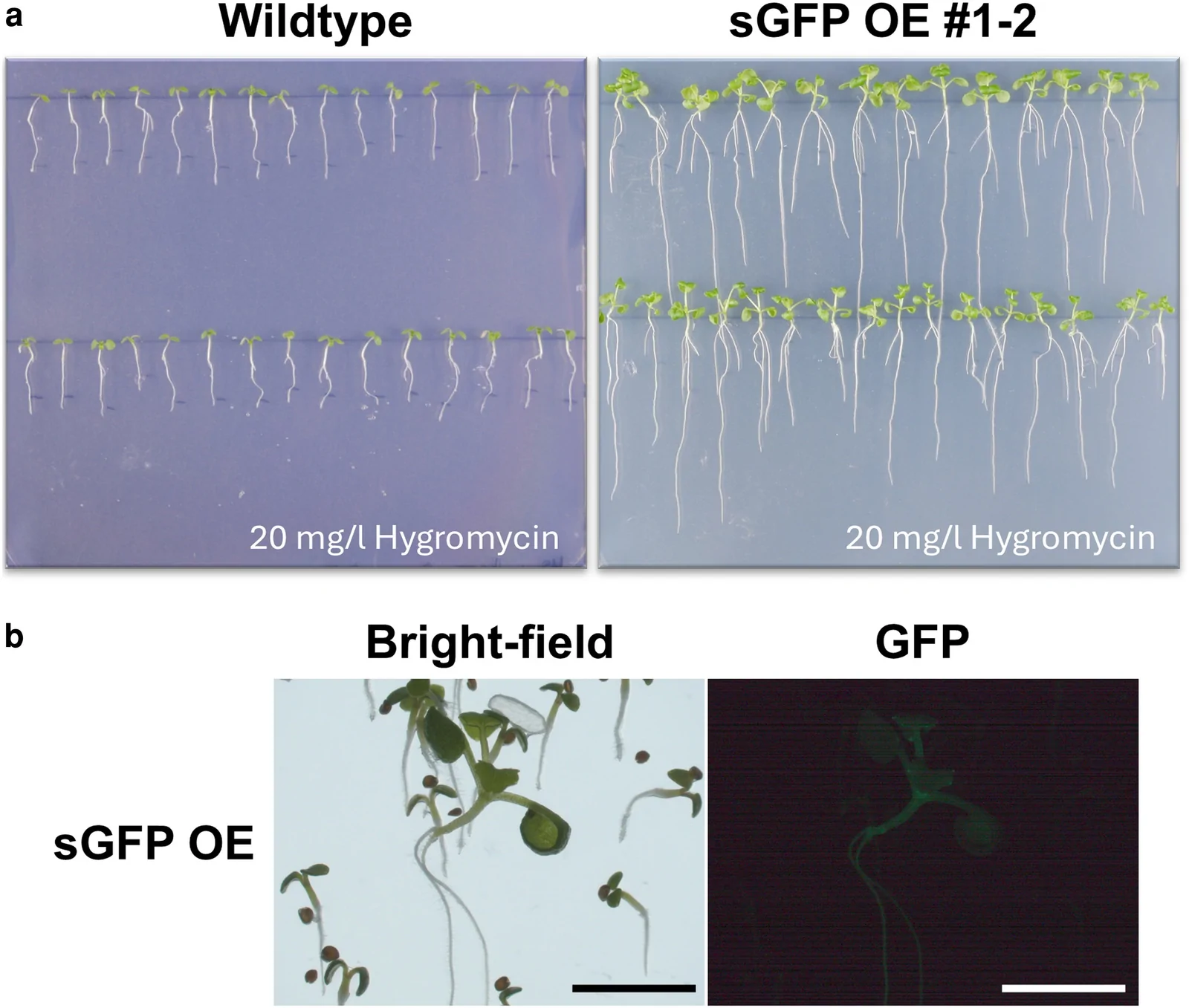
Transformation of submergence-tolerant *R. islandica*. (a) Growth of seedlings on 20 mg/L hygromycin (wild type left, synthetic GFP overexpressor line right). (b) Successful transformant containing the construct pMDC32-GFP on 20 mg/L hygromycin under white light (left) and fluorescence light (right). Bar = 5 mm. See online Supplementary material for a color version of this figure.

Arabidopsis and *C. hirsuta* are established model systems, and both can be transformed by the easy floral dip method (Hay et al. 2014). We therefore hypothesized that *R. islandica* could be transformed by this method as well. To test this, we first used a plasmid containing the coding sequence of GFP and a gene conferring hygromycin resistance. Indeed, the floral-dip method resulted in a small number of positive transformants with hygromycin resistance that also showed green fluorescence (Fig. 3). Transgenic plants could be easily propagated and made homozygous through selfing. However, overall seed germination and transformation rates were very low in this species, but the high number of seeds produced still allowed for further experiments.

### *BCA3* expression is up-regulated by submergence-induced ethylene

**Figure 4:**
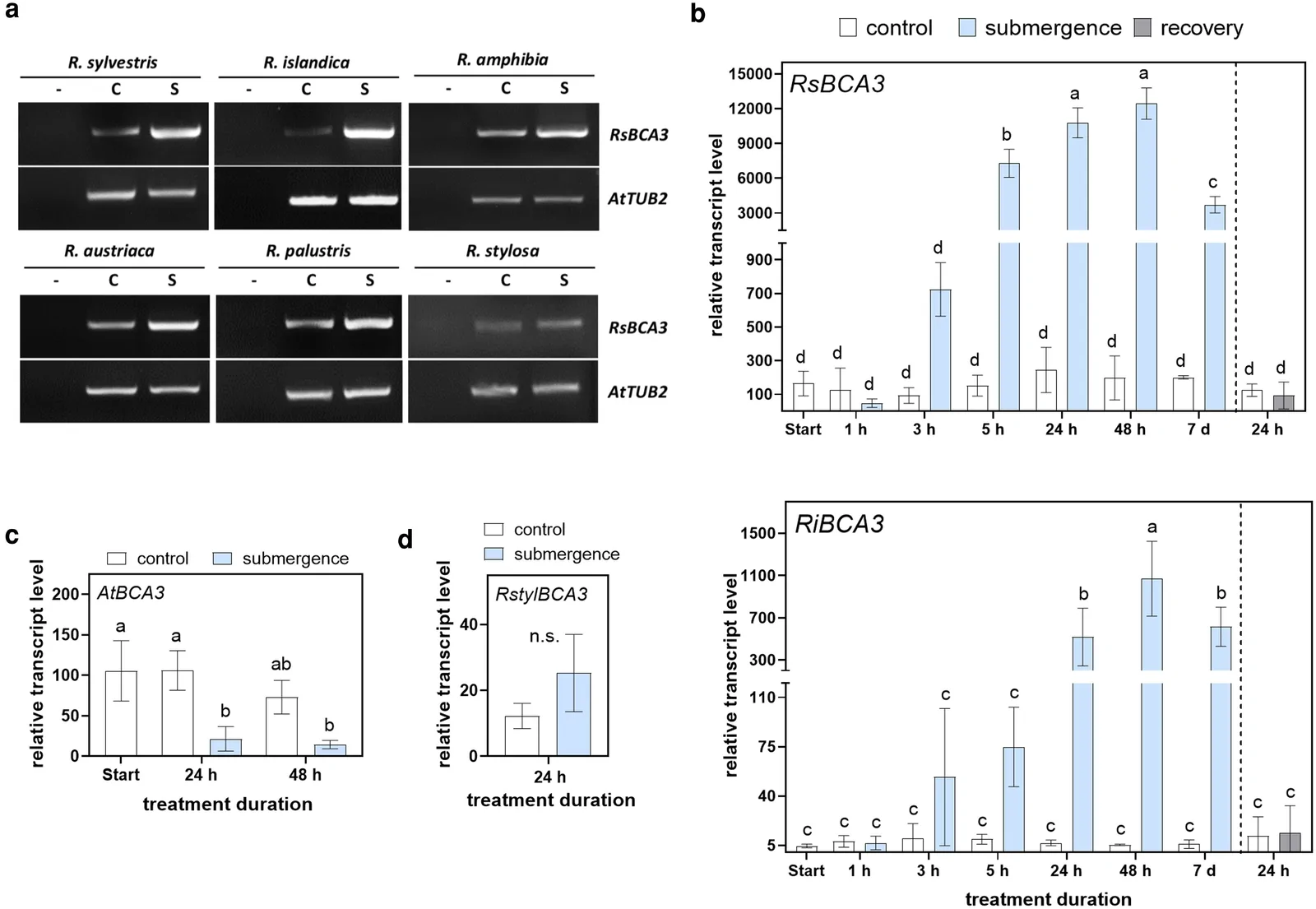
*BCA3* is induced by submergence in tolerant *Rorippa* species. (a) Reverse transcription PCR of *RsBCA3* and *AtTUB2* in 6 *Rorippa* species. (b) Reverse transcription quantitative PCR of *RsBCA3* and *RiBCA3* after different durations of submergence. Data were normalized to *TUB6* and *ACT2*. (c) Reverse transcription quantitative PCR of *AtBCA3* after different durations of submergence. Data were normalized to *PP2A* and *UBC9*. (B and C) Data are means ± SD from 3 independent replicates. Different letters indicate significant differences (2-way ANOVA, Tukey honestly significant difference test, *P* < 0.05). (d) Reverse transcription quantitative PCR of *RstylBCA3* after 24 h of submergence. Data were normalized to *TUB6* and *ACT2*. Data are means ± SD from 3 independent replicates. No significant differences were observed between treatment types (Student *t*-test, *P* < 0.05).

Among the 196 genes with an overall high expression in submerged *R. islandica* samples (base mean > 500), there was the gene *BCA3*, supporting our earlier findings on other *Brassicaceae* species (Dataset S4). The high expression of the *BCA3* gene in the tolerant plant species *R. sylvestris* (van Veen et al. 2024) and *R. islandica* (Figure S5), but not in sensitive *Arabidopsis* or *C. hirsuta*, as well as its potential association with low CO_2_ levels under water (Figure S6), motivated us to select this as a candidate gene for further studies. In a first experiment, we verified its expression patterns in different plant species. Reverse transcription PCR analysis revealed that the gene expression was induced by submergence in 5 out of 6 *Rorippa* species, while it was not expressed higher in sensitive *R. stylosa* (Fig. 4a). Time-series expression analyses via reverse transcription quantitative PCR showed a significant increase in expression after 5 h of submergence in *R. sylvestris*, and after 24 h in *R. islandica* (Fig. 4b). Expression levels remained high throughout a 7-day submergence period and decreased again within 24 h of reaeration. In Arabidopsis and *R. stylosa*, *BCA3* expression was not significantly increased during submergence (Fig. 4c and d).

**Figure 5:**
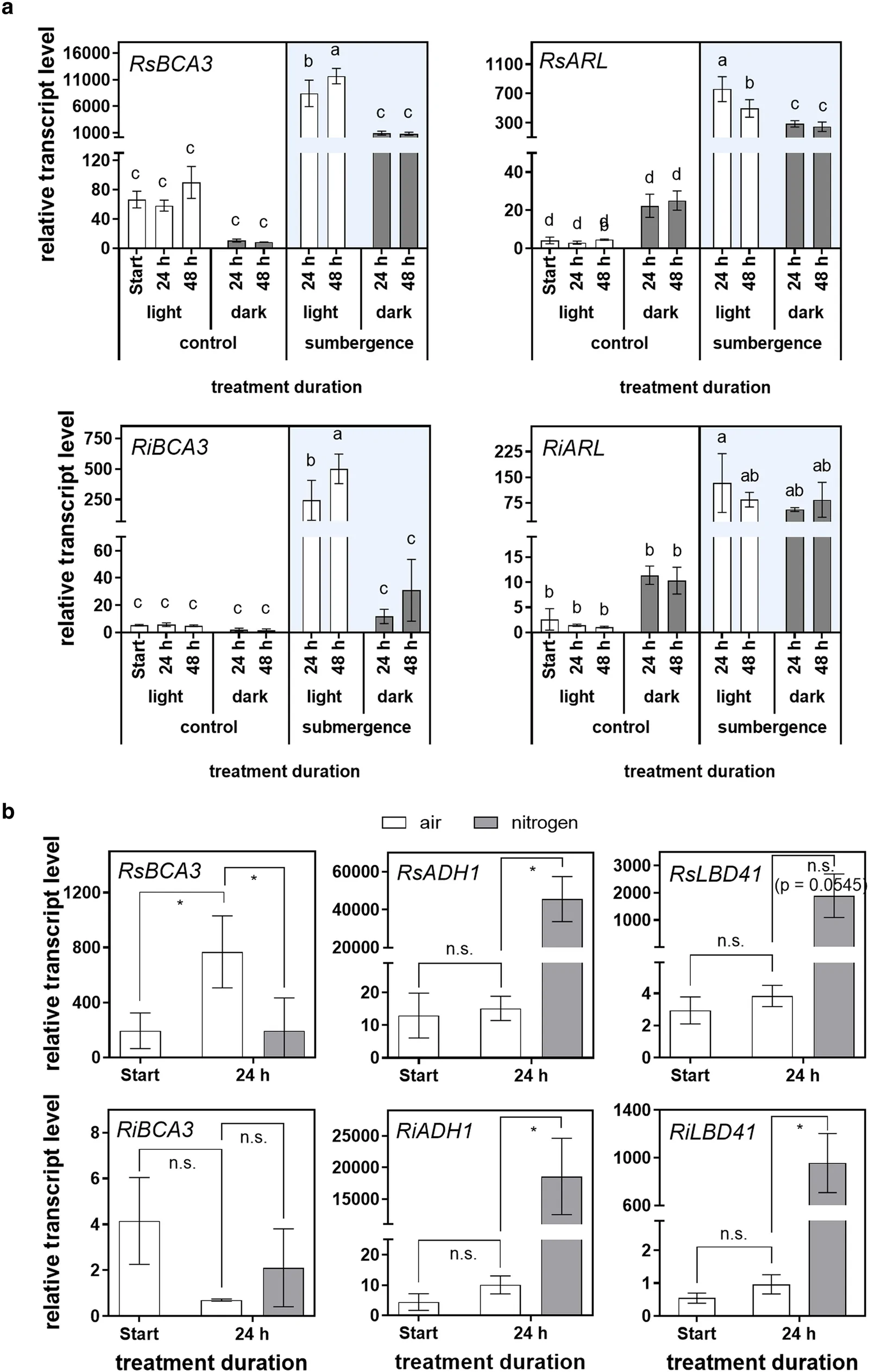
Impact of light and hypoxia on *BCA3* expression. (a) Reverse transcription quantitative PCR of *BCA3* and *ARL* after different durations of submergence in light and darkness. Data were normalized to *TUB6* and *ACT2*. (b) Reverse transcription quantitative PCR of *BCA3*, *ADH1*, and *LBD41* after 24 h of hypoxia. Data were normalized to *PP2A* and *UBC9*. (A and B) Data are means ± SD from 3 independent replicates. Different letters indicate significant differences (2-way ANOVA, Tukey honestly significant difference test, *P* < 0.05).

We then considered the environmental factors that are responsible for gene induction, selecting the diploid *R. islandica* and tetraploid *R. sylvestris* for further analyses. Submergence has multiple stress-related components—namely, reduced gas exchange leading to low oxygen and CO_2_ concentrations as well as increased in-planta ethylene content. Another factor might be the lower light level underwater. First, we tested whether illumination influences *BCA3* expression. Again, 24 and 48 h of submergence in illumination caused a strong and significant increase in *BCA3* expression in both species (Fig. 5a). In dark submergence, *BCA3* levels only increased slightly, compared with aerated controls, and this difference was not significant.

To test the impact of hypoxia, we gassed *Rorippa* plants with nitrogen gas, mimicking a treatment that had been often used for Arabidopsis seedlings (eg, Mustroph et al. 2009). This stress treatment caused the expected upregulation of 2 hypoxia-responsive core genes, *ADH1* and *LBD41* (Mustroph et al. 2009), but not of *BCA3* (Fig. 5b). In addition, this experiment also ruled out CO_2_ deficiency as a regulating factor since the atmosphere also was free of this gas. Carbon dioxide availability was further tested by submerging the plants in water with different pH ranging from 5.0 (high CO_2_) to 8.0 (low CO_2_) since soluble CO_2_ is strongly dependent on pH (Maberly and Spence 1983; Maberly and Gontero 2017). In each submergence treatment, *BCA3* was strongly induced, independent of the pH of the flood water (Figure S9).

**Figure 6:**
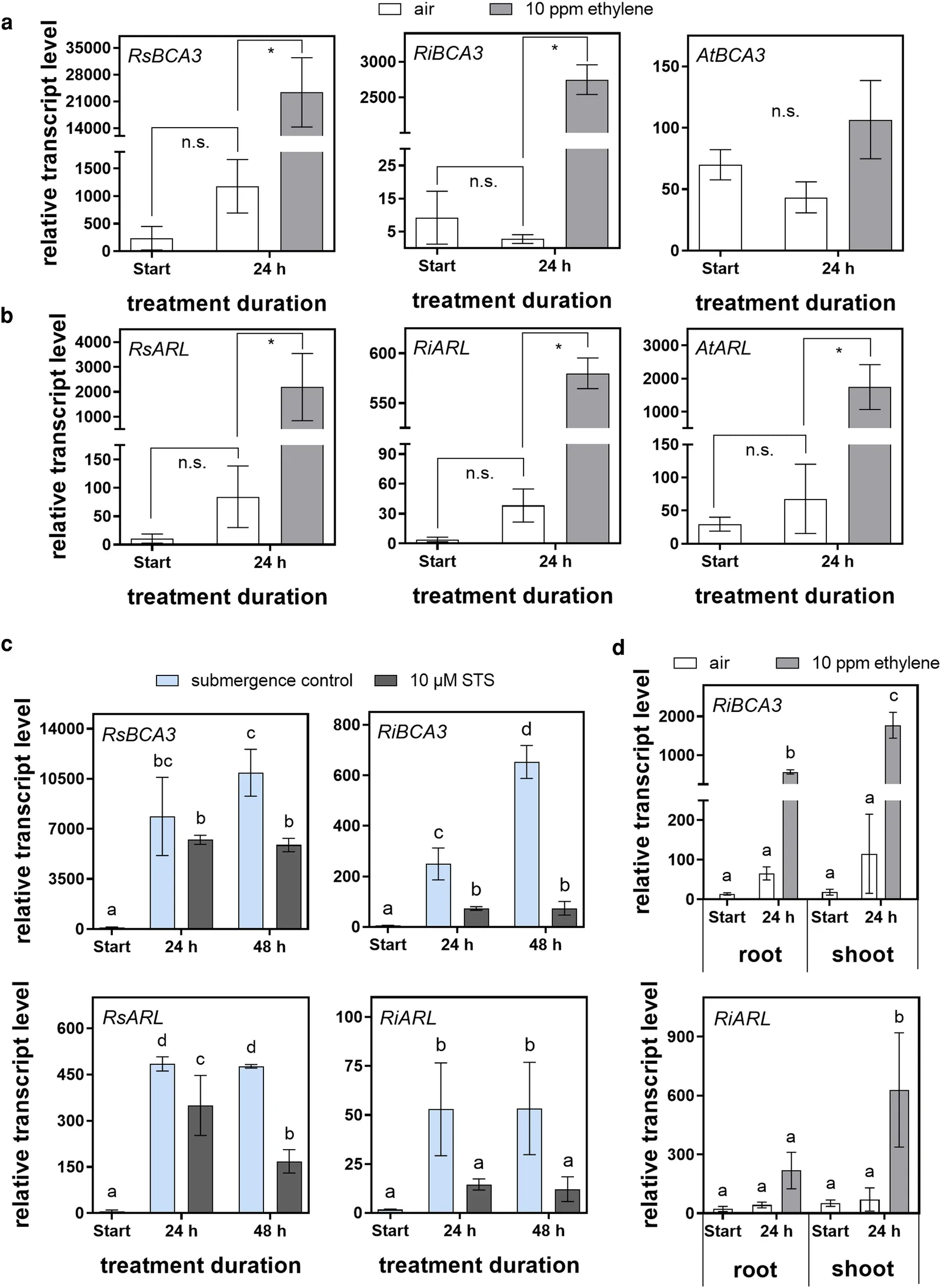
*BCA3* induction is dependent on ethylene. (A and B) Reverse transcription quantitative PCR of *BCA3* (a) and *ARL* (b) 24 h after treatment with 10 ppm ethylene in leaves of *R. sylvestris*, *R. islandica*, and *A. thaliana*. (c) Reverse transcription quantitative PCR of *BCA3* and *ARL* after different durations of submergence with the ethylene biosynthesis inhibitor silver thiosulfate. (d) Reverse transcription quantitative PCR of *RiBCA3* and *RiARL* 24 h after treatment with 10 ppm ethylene in seedlings of *R. islandica*, separated into roots and shoots. (a-d) Data were normalized to *TUB6* and *ACT2* for *Rorippa* species, and to *PP2A* and *UBC9* for *A. thaliana*. Data are means ± SD from 3 independent replicates. Different letters indicate significant differences (2-way ANOVA, Tukey honestly significant difference test, *P* < 0.05).

The last and most likely option was ethylene accumulation under water, indicated also by our transcriptome analysis (Dataset S1, Figure S3). Indeed, we confirmed that ARGOS-LIKE (*ARL*), a gene with known responsiveness to ethylene in Arabidopsis (Rai et al. 2015) and high expression in all plant species under submergence (Dataset S3), was also strongly induced under submergence in both species in light and darkness (Fig. 5a). Exposing *Rorippa* and Arabidopsis plants to 10 ppm ethylene indeed caused a significant upregulation of *ARL* as well as *BCA3* (Fig. 6a and b). In *R. islandica* seedlings, this upregulation was higher in shoots compared with roots, but still significant in both organs (Fig. 6d). In addition, the ethylene biosynthesis inhibitor silver thiosulfate significantly reduced the submergence-induced increase in *BCA3* and *ARL* expression (Fig. 6c). However, this effect was more pronounced in *R. islandica*, potentially related to a lower diffusion rate of the chemical into *R. sylvestris* leaves.

### *BCA3* as a candidate gene for submergence tolerance

Having established that *BCA3* is strongly expressed in submergence-tolerant *Rorippa* plants, we wanted to explore whether it contributes to the high submergence tolerance and what function it might fulfill under submergence. First, we used a yeast expression system to verify its activity as a CA (Rai et al. 2022). Expression of *BCA3* from 3 species (*R. islandica*, *R. sylvestris*, and Arabidopsis) rescued a yeast mutant defective in CA activity (Figure S10).

**Figure 7:**
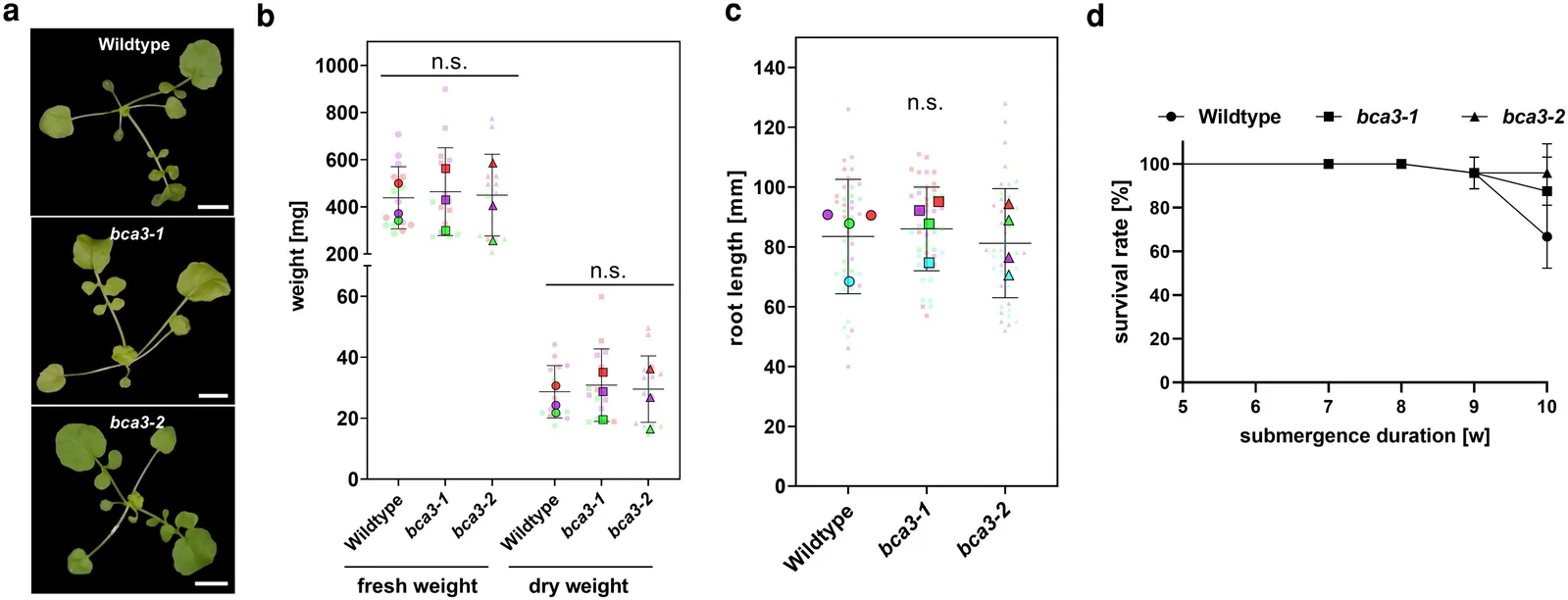
Phenotype of *R. islandica bca3* CRISPR-based knockout plants. (a) Photographs of 4-week-old plants. Images were digitally extracted for comparison. Bar = 1 cm. (b) Fresh and dry weight of 3-week-old plants. n = 15 from 3 independent experiments. (c) Root length of 10-day-old seedlings on Hoagland medium. n = 44 to 48 from 4 independent experiments. (B and C) Data are means ± SD. Independent experiments are marked with different colors. Different symbols mark different genotypes. (d) Survival after 7 to 10 weeks of submergence in a day–night rhythm. Survival was defined as the ability to produce new leaves within the recovery phase under normal growth conditions for 2 weeks. Data are means ± SD from 3 independent experiments with 8 plants per replicate and time point. (B and C) Different letters indicate significant differences (1-way ANOVA, Tukey honestly significant differences test, *P* < 0.05). See online Supplementary material for a color version of this figure.

Next, we created CRISPR-Cas9–mediated *R. islandica* knockout lines for *RiBCA3*. Two independent lines with a frameshift in the coding sequence led to an early stop codon and a nonfunctional protein based on yeast complementation assays (Figure S11). An in-depth analysis of the transgenic lines revealed no growth defects of the 2 *bca3* mutants at the adult (Fig. 7a and b) or seedling stage (Fig. 7c). Surprisingly, both mutant lines had similar survival rates under submergence compared with the wild type (Fig. 7d). Next, we tested whether reduced *BCA3* expression and activity influence the expression of the other *BCA* genes. None of the tested transcripts were altered due to the mutation, besides *BCA3* itself (Figure S12). Nevertheless, submergence caused induction of *RiBCA2* and *RiBCA3*, as well as *RiBCA6*, confirming the RNA sequencing (RNA-seq) data (Dataset S1), while *RiBCA1*, *RiBCA4*, and *RiACA1* were significantly downregulated.

To assess changes due to missing BCA3 activity at the biochemical level, further tests were performed. Our initial hypothesis was that BCA3 might be involved in providing CO_2_ for photosynthesis, also supported by the observation that *RiBCA3* was only induced in illuminated submergence (Fig. 5a). However, no significant changes in photosynthesis, based on oxygen evolution, were observed between genotypes, but a slight increase in underwater photosynthesis was measured for plants that had already acclimated to submergence for 48 h (Figure S13). Similarly, there were no differences in sugar contents between genotypes with and without submergence, but a general, strong decrease in sugar levels after submergence (Figure S14).

CAs could also be involved in other biochemical processes that require HCO^3–^ (eg, fatty acid biosynthesis or amino acid metabolism) (Nikolau et al. 2003; Aguilera et al. 2005). However, among 18 amino acids, none was modified in its content in lines with reduced BCA3 activity compared with the wild type (Figure S15). On the other hand, amino acids dramatically changed under submergence. Levels of arginine, glycine, and serine increased significantly, while alanine, asparagine, glutamine, and aspartic acid levels decreased upon submergence. Similarly, fatty acids were not significantly different in the 3 genotypes, with a slight decrease in C16:1 and C18 fatty acids during submergence (Figure S16).

**Figure 8:**
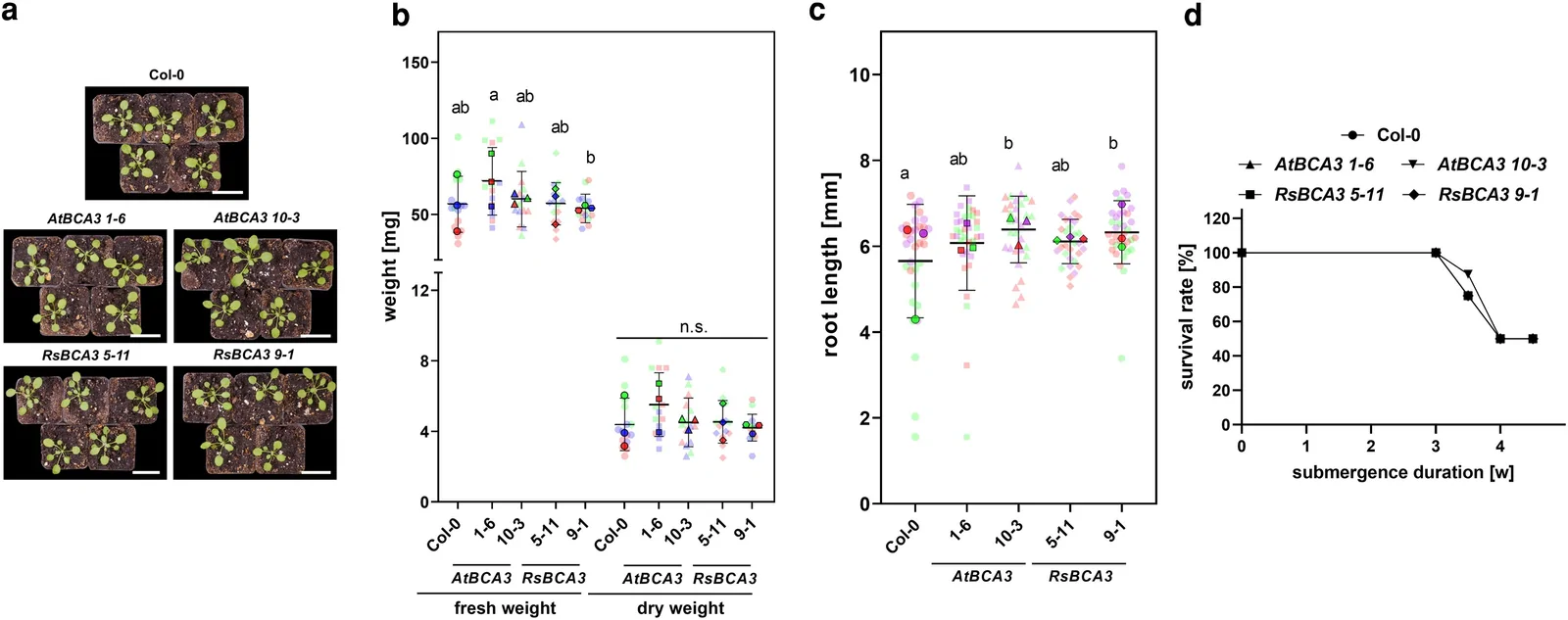
Phenotype of *A. thaliana* lines overexpressing *AtBCA3* or *RsBCA3*. (a) Photographs of 4-week-old plants. Images were digitally extracted for comparison. Bar = 3 cm. (b) Fresh and dry weight of 3-week-old plants. n = 15 from 3 independent experiments. (c) Root length of 7-day-old seedlings on MS medium. n = 31 to 35 from 3 independent experiments. (b and c) Data are means ± SD. Independent experiments are marked with different colors. Different symbols mark different genotypes. (d) Survival after 3 to 5 weeks of submergence in a day–night rhythm. Survival was defined as the ability to produce new leaves within the recovery phase under normal growth conditions for 2 weeks. Data are means ± SD from 3 independent experiments with 8 plants per replicate and time point. (B and C) Different letters indicate significant differences (1-way ANOVA, Tukey honestly significant differences test, *P* < 0.05). See online Supplementary material for a color version of this figure.

A possible function of BCA3 was further tested by overexpressing it in Arabidopsis plants. Both *AtBCA3* and *RsBCA3* were expressed under the control of the 35S promoter (Figure S17). The N-terminal HA tag did not impact BCA activity, as demonstrated in the yeast system (Figure S10) and in previous experiments (Rai et al. 2022). Again, higher *BCA3* expression did not influence the growth of adult plants (Fig. 8a and b) or seedlings (Fig. 8c). Furthermore, there was no difference in submergence survival between the different Arabidopsis lines (Fig. 8d).

### Photosynthesis is associated with submergence tolerance

**Figure 9:**
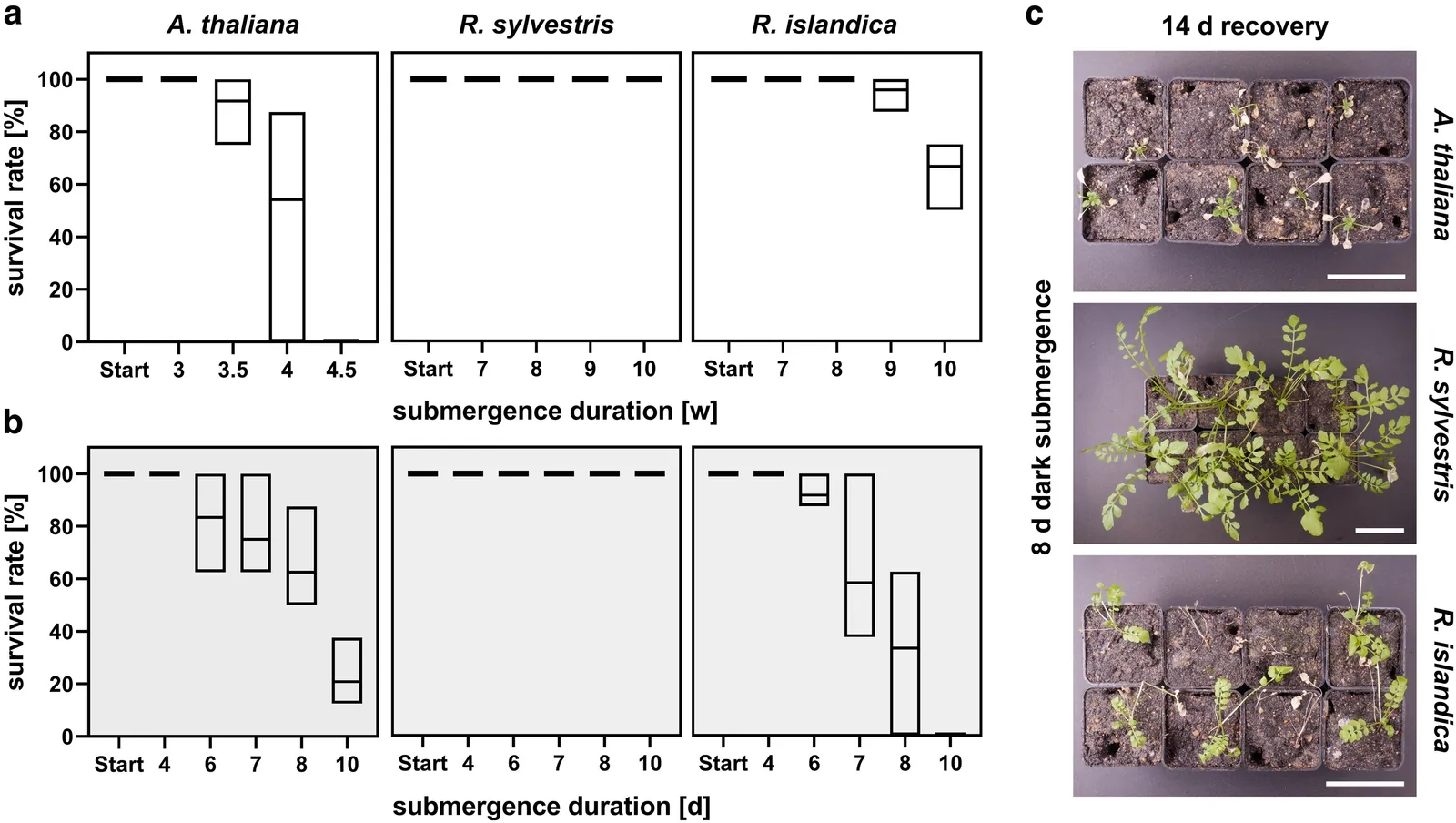
Submergence tolerance of *R. islandica* is dependent on illumination. (a) Survival rate of Arabidopsis and *Rorippa* plants subjected to submergence for 3 to 10 weeks in a day–night rhythm and subsequently recovered under normal growth conditions for 2 weeks. (b) Survival rate of Arabidopsis and *Rorippa* plants subjected to submergence for 4 to 10 days in darkness and subsequently recovered under normal growth conditions for 2 weeks. (c) Photographs of plants after 8 days of dark submergence and 14 days of recovery. Bar = 5 cm. (A and B) Survival was defined as the ability to produce new leaves. Data are means ± SD from 3 independent experiments with 8 plants per replicate and time point.

The previously described experiments did not reveal an important role for BCA3 in submergence tolerance. Therefore, other factors might be responsible for high submergence tolerance. The 3 species (Arabidopsis, *R. islandica*, and *R. sylvestris*) were used for further experiments that might help to elucidate the submergence tolerance. First, it was tested whether the difference in submergence tolerance was dependent on illumination—that is, whether factors associated with photosynthesis should be explored further. The very low submergence tolerance of Arabidopsis compared with both *Rorippa* species under illumination was indeed less pronounced in darkness. *R. islandica* did not show better survival in dark submergence compared with Arabidopsis (Fig. 9). However, both species benefited from illumination during submergence. In contrast, *R. sylvestris* was also more tolerant to dark submergence compared with the other 2 species. Whether this tolerance was related to the perennial lifestyle of this species needs to be studied further.

Besides oxygen and ATP production, photosynthesis could lead to higher carbohydrate production, which could be responsible for higher submergence tolerance, as previously suggested (van Veen et al. 2024). However, while soluble carbohydrate levels in *R. sylvestris* were again slightly higher compared with the other 2 species, there was no difference between the 2 annual species, Arabidopsis and *R. islandica*, at the time points analyzed (Figure S18). Moreover, most genes associated with photosynthesis were not differentially expressed under submergence or were downregulated in *R. islandica* (light harvesting complexes) (Datasets S1 and S6). Only a small set of genes was upregulated at the transcriptional level, including *AT4G14690* (*ELIP2*), *AT3G22840* (*ELIP1*), and *AT2G21970* (*SEP2*). *ELIP2* is among the 196 candidate genes that are more highly expressed in tolerant *R. islandica* compared with *R. stylosa* (Dataset S4).

## Discussion

### *Rorippa islandica* is a genetically accessible model organism for flooding tolerance

The genetically accessible model organism *Arabidopsis thaliana* has a relatively low flooding tolerance, although some natural variation exists (Vashisht et al. 2011; van Veen et al. 2016). The only established model plant from the flooding-tolerant Cardamineae tribe, *Cardamine hirsuta*, has a higher flooding tolerance compared to Arabidopsis, but only at a moderate level, while other species of this tribe show very high levels of tolerance, especially from the genus *Rorippa* (van Veen et al. 2024). Because the most tolerant species from the previous study were polyploid and without published genomes, we extended our previous analyses to 2 diploid *Rorippa* species, *R. islandica* and *R. stylosa* (*R. pyrenaica*). While *R. stylosa* shows a rather low submergence tolerance, *R. islandica* has a survival rate of 9 to 10 weeks of submergence (Figs. 1 and 7), close to the best-performing tetraploid *Rorippa* species (Sasidharan et al. 2013; van Veen et al. 2024).

Therefore, we considered *R. islandica* an ideal candidate for the establishment of a model species for flooding tolerance. Its diploid nature, its ability to produce many seeds through selfing, its relatively short generation time, and the independence of flowering from vernalization were further positive factors of this species. In addition, its genome sequence is publicly available. Here we demonstrate the successful transformation of this species via the floral-dip method using a GFP-coding plasmid (Fig. 3), like *C. hirsuta* (Hay et al. 2014). Moreover, transformation with a CRISPR-Cas9 vector was also successful, and positive gene editing was observed in the T2 and T3 generations (Figure S11a). Therefore, this species is an ideal candidate to study flooding tolerance in the Brassicaceae family.

### The submergence response of Brassicaceae

Both species in this analysis as well as all species from the previous studies (Müller et al. 2021; van Veen et al. 2024) show a strong transcriptome modulation following 24 and 48 h of submergence, indicated by the number of DEGs (Fig. 2b). In the direct comparison between the 2 species used here there was a higher number of DEGs in tolerant *R. islandica* compared with sensitive *R. stylosa* (Fig. 2), but this trend cannot be generalized and was not observed in other species, since the number of differentially expressed OGs in sensitive Arabidopsis was as high as that of tolerant *Rorippa* species (van Veen et al. 2024).

The overlap between DEGs was rather high; about 50% of transcripts were regulated in both species (Fig. 2c). This also holds true when the 2 species were included in our previous dataset (van Veen et al. 2024), demonstrating differential expression of diverse OGs in most species (Dataset S2). Among commonly regulated genes, only a few were among the previously defined hypoxia-response genes (eg, *HUP36/RGAT1*, *CML38*, *ETR2*, *PP2-A13*, *HUP54*, *ACHT5*) (Figure S1) (Mustroph et al. 2009). Interestingly, many of those submergence-induced hypoxia-response genes have been recently suggested not to be specific hypoxia marker genes but are rather induced by carbon starvation or ethylene (Renziehausen et al. 2024). This is in accordance with in planta measurements demonstrating oxygen production through photosynthesis during submergence (in light) (eg, Lee et al. 2011; Müller et al. 2021; Wittig et al. 2021). Furthermore, in this analysis (Figures S14 and S18) as well as in previous studies, submergence led to a severe depletion of carbohydrates within a few hours (eg, Yeung et al. 2018; Wittig et al. 2021; Müller et al. 2021; van Veen et al. 2024), accompanied by a strong induction of carbon starvation marker genes (Figure S2) previously identified in Arabidopsis (Usadel et al. 2008; Cookson et al. 2016; Renziehausen et al. 2024). This trend had also been observed for other Brassicaceae species in our earlier studies (Müller et al. 2021; Wittig et al. 2021; van Veen et al. 2024).

In addition, ethylene is an early marker for submergence (ie, reduced gas exchange) and in our previous analysis as well as here, many ethylene-responsive genes (Goda et al. 2008; Harkey et al. 2018; Liu et al. 2022; Renziehausen et al. 2024) were also induced by submergence (Figure S3). This confirms the important role of ethylene as a signaling molecule under submergence stress (summarized in Voesenek and Sasidharan 2013; Sasidharan et al. 2018; Renziehausen et al. 2024), as exemplified by *RiBCA3* regulation (see the following discussion) (Fig. 6). However, other signaling events can be involved in the regulatory process in some species since inhibition of ethylene biosynthesis through silver thiosulfate did not completely abolish *RsBCA3* induction in *R. sylvestris* (Fig. 6). A similar effect had been observed for submergence-induced stem elongation in *N. officinale*, where other signaling pathways besides ethylene were possibly involved (Müller et al. 2021). Other potential signals could be related to low light availability under water or related to carbon or CO_2_ starvation, but this requires further analyses.

These similarities in gene expression hint at a common transcriptome response of Brassicaceae plant species to submergence. These common changes might enable conserved acclimation responses for submergence survival for up to 3 weeks, but they are most likely not responsible for enhanced tolerance of *R. islandica* or *R. sylvestris*. Indeed, the hypoxia response also has similarities between several unrelated plant species, as previously shown (Mustroph et al. 2010). The hypoxia-response signaling pathway through group VII ethylene response factors is conserved across plant species (van Veen et al. 2014; Lin et al. 2019; Taylor-Kearney et al. 2022; Weits et al. 2023), and it is likely that the submergence response, which is somewhat different from sole hypoxia, is also commonly regulated at the transcriptome level.

While the ethylene and carbon starvation responses were conserved across Brassicaceae species, there were also some species-specific responses. Immunity-related processes were among enriched GO terms mainly in *R. islandica* (Dataset S1), which might hint at a better preparation for pathogen infection during and after submergence. The floodwaters might contain more microorganisms mobilized from the soil, providing an increased threat for the plants. In addition, the low-energy status of the plants negatively impacts plant defense responses. An involvement of the pathogen response under submergence has been suggested for Arabidopsis (Hsu et al. 2013a; Brunello et al. 2024). However, *R. stylosa*, which is also sensitive, can induce pathogen-related processes, namely camalexin biosynthesis.

Another specific response was observed among downregulated genes. In *R. islandica*, several genes associated with stomata development were reduced upon submergence, but not in *R. stylosa* (Figure S4, Dataset S1). Indeed, for a related species, *R. aquatica*, heterophylly was observed under submergence, leading to more dissected leaves with fewer stomata underwater, thereby enhancing gas diffusion through the cuticle to support photosynthesis (Ikematsu et al. 2023). Although our sampling point was too early to include newly formed leaves, this modified gene expression might hint at a similar acclimation response in *R. islandica*. Similarly, the 2 *Rorippa* species from our earlier study showed the same tendency (van Veen et al. 2024).

Despite the massive changes in gene expression caused by submergence, there was no clear trend in modification of photosynthesis, besides a reduced expression of light-harvesting complexes in *R. islandica* (Dataset S6), and increased expression of low-light responses (Dataset S1). However, photosynthesis is mainly regulated at the posttranslational level, which we did not analyze here. We therefore cannot exclude modifications of the photosynthetic process in submerged plants. Other plant species showed specific modifications of photosynthesis-related processes such as uptake and conversion of bicarbonate, C4- or CAM-like photosynthesis (eg, Keeley 1990; Klavsen et al. 2011; Keeley 2014; Maberly and Gontero 2017; Yin et al. 2017; Iversen et al. 2019), but there was no evidence for any of these responses in any Brassicaceae species when focusing on expression of genes that had been associated with these processes (Figure S6; Dataset S2) (Goolsby et al. 2018; van Veen et al. 2024). For example, none of the plant species tested here was able to perform photosynthesis at pH 8.3, a condition where CO_2_ is no longer present but transformed into HCO_3_^−^ (Figure S13).

### RiBCA3 has no essential role under submergence

Having a transformable plant species with high submergence tolerance in hand, we selected a target gene for molecular analysis as a test case. Since CO_2_ availability under water is severely lowered and considerably affects photosynthesis, as demonstrated here by low carbohydrate production (Figures S14 and S18), we assumed that plants could compensate for this lack of CO_2_ with the enhanced expression of CAs that reversibly catalyze the transformation of CO_2_ to HCO_3_^−^. Indeed, *R. islandica* and *R. sylvestris* had a strongly induced expression of *BCA3* under submergence, with a very high overall expression level, while in sensitive Arabidopsis this gene was not induced, and induction in *R. stylosa* was only mild (Fig. 4; Datasets S1 and S2).

First, we asked which signals were involved in its upregulation under submergence. As indicated in the following discussion, plants under water experience different modifications (ie low oxygen and low CO_2_ availability, low light levels, ethylene enrichment). Among the tested factors, neither low light, low oxygen, nor low CO_2_ concentrations increased *BCA3* gene expression (Fig. 5a and b, Figure S9). As previously mentioned, submerged plants do not encounter severe hypoxia during illuminated hypoxia (Lee et al. 2011; Müller et al. 2021; Wittig et al. 2021; Panicucci et al. 2024); therefore, it is not surprising that hypoxia alone does not cause *BCA3* induction. However, our experiments clearly demonstrated that ethylene was involved in the induction of *BCA3* expression, as ethylene treatment itself caused a similar response, and inhibition of ethylene biosynthesis inhibited *BCA3* expression under submergence (Fig. 6). This result suggests that ethylene might be a major player in the transcriptional response of plants to submergence.

Second, we analyzed a possible function of *RiBCA3* under control or submergence conditions, through knockout lines in *R. islandica* (Figure S11) and overexpression lines in Arabidopsis (Figure S17). However, no differences in growth, submergence survival, photosynthesis, or metabolism were discovered between genotypes (Figs. 7 and 8; Figures S13–S16). In addition, loss of *RiBCA3* was not complemented by any other *BCA* gene at the transcript level (Figure S12). We therefore concluded that BCA3 alone has no important role for submergence survival of *Rorippa* species. However, we cannot rule out that other functions of BCA3 that were not analyzed here might be present. In addition, there could be the possibility that the loss of *RiBCA3* can be compensated for by other BCAs, which are still expressed and could even be regulated at the posttranslational level. For example, *RiBCA2* and *RiBCA6* are also induced under submergence, but at a lower level (Figure S12). Moreover, since no bicarbonate transporter was identified among the submergence-induced genes, Brassicaceae plants might not be able to take up HCO_3_^−^ from the floodwater. In general, CO_2_ uptake over membranes is higher than for HCO_3_^−^ if no specific transporter is present (Gutknecht et al. 1977).

### Anatomy and underwater photosynthesis as future research directions in the model system *R. islandica*

Although our experiments did not reveal a significant role of *RiBCA3* in submergence tolerance, the data still present a useful dataset. On one hand, they clearly demonstrate that *R. islandica* is a genetically accessible model system. This system can be used to study other relevant genes from the dataset, as well as transcriptional regulators to evaluate acclimation responses in general. Furthermore, forward genetic screens after undirected mutagenesis through ethyl methanesulfonate could reveal important factors for plant tolerance. On the other hand, many changes in response to submergence have been observed, not only at the level of transcription (Dataset S1; Fig. 2), but also at the level of metabolism (Figures S14–S16) and photosynthesis (Figure S13). These changes can be used to develop hypotheses on molecular mechanisms of flooding tolerance, which then could be tested through transgenic plants.

One of these mechanisms could involve anatomical features in leaves (see the previous discussion) as well as roots. Indeed, *R. islandica* and *R. sylvestris* can form wheel-shaped schizogenous aerenchyma in roots (Figure S19), a phenomenon that had not been observed before for this genus (Jung et al. 2008) but had been recently described for *Cardamine amara* (Kudoh et al. 2025). Gas spaces in petioles of *Rorippa* have been previously observed (Akman et al. 2012), and modifications in the epidermis and leaf shape are likely (Ikematsu et al. 2023). To study anatomical changes, stress treatments should be extended at least to 1 to 2 weeks. Indeed, while sensitive Arabidopsis and *R. stylosa* were not able to form new leaves under water, *R. islandica* and *R. sylvestris* were able to do so. Furthermore, roots need to be analyzed, including cell-type or tissue-specific analyses.

Another direction could be photosynthesis, although we have only observed minor changes in the expression of genes related to photosynthesis (Dataset S6). However, there was a tendency of enhanced underwater photosynthesis after a 48-h pretreatment with submergence in *R. islandica* and *R. sylvestris* (Figure S13). In addition, *R. islandica* was only more tolerant than Arabidopsis under illuminated submergence, but not under dark submergence (Fig. 9), suggesting that illumination was responsible for the enhanced submergence tolerance between these 2 annual species. According to expression data, *ELIP2* could be a potential tolerance-associated target for future studies (Datasets S4 and S6). However, since photosynthesis is mainly regulated posttranslationally (Giese et al. 2023), other approaches like proteomics combined with metabolomics are required to study the underlying mechanisms.

Another route of research to reveal underlying tolerance mechanisms could be at the genomic level. Our analysis of sequence variations, based on expressed genes, demonstrated that about 95% of all genes in *R. stylosa* had small modifications compared with the published genome of *R. islandica*, as expected for 2 separate species (Figure S8, Dataset S5). This makes it difficult to narrow down candidate genes responsible for submergence tolerance. Furthermore, the sequence data cover only part of the *R. stylosa* genome. A full genome sequence of *R. stylosa* is required to dig further into this topic, including promoter sequences, and the possibility to evaluate copy-number variations.

### Conclusions

With this work, we could confirm that species of the Cardamineae tribe of the Brassicaceae family have very high submergence tolerance. However, this is not true for all species of this tribe, as shown previously for *Cardamine hirsuta* and now for *Rorippa stylosa*. Therefore, each species must be separately analyzed in respect to submergence tolerance. Here, *Rorippa islandica* was identified as a species with high submergence tolerance. In addition, it can be transformed and is therefore a suitable model system for future studies to decipher the molecular mechanisms of flooding tolerance in dicotyledonous species, as rice is for monocotyledonous species. In nature, this species has a relatively narrow distribution range, restricted to Eurasian mountain regions. However, its relatedness to crops within the Brassicaceae family, such as rapeseed and cabbage, might allow for a transfer of knowledge obtained for this species into agriculture. The mechanisms of submergence tolerance, however, were not yet revealed within the frame of this study. The candidate gene *RiBCA3* seemed to be not involved in submergence tolerance. Further work is therefore required, using this model species, to decipher the most important factors for tolerance. An involvement of light and photosynthesis is likely. A deeper comparison of the transcriptomic response of sensitive and tolerant species in this tribe might reveal specific target genes for future experiments.

## Supplementary Material

kiag254_Supplementary_Data

## Data Availability

The data underlying this article are available in the article and in its online supplementary material, and in NCBI’s Gene Expression Omnibus database (GEO accession GSE295090).
