# Supplementary material for: Rorippa islandica: a genetically accessible dicot model system to study flooding tolerance
Source: Plant Physiol. 2026 Apr 29;202(1):kiag254. doi: 10.1093/plphys/kiag254 (PMC13591404; doi:10.1093/plphys/kiag254)
Supplement: Supplementary file 1 [file kiag254_supplementary_data.zip › PLPHYS-2025-1197R1_Supplement.pdf]

The discovery of flooding-resistant *Rorippa islandica* enables the establishment of a genetically accessible dicot model system flooding tolerance

Malte M. Bartylla, Emma L.R. Düthorn, Jana T. Müller, Markus Wirtz, Hans van Veen, Rashmi Sasidharan, Angelika Mustroph

SUPPLEMENTARY MATERIALS

Supplementary Figures

-3 0 3 log_2FC

R. stylosa 24 h	*R. stylosa* 48 h	*R. islandica* 24 h	*R. islandica* 48 h	Rorippa-ID	AGI	Annotation
NA	NA	NA	NA	NA	AT1G17290	ALAAT1
8.72	10.11	6.99	7.61	Roisl.0007s0177	AT1G19530	RGAT1
1.5	1.68	2.25	1.99	Roisl.0191s0386	AT1G26270	*Phosphatidylinositol 3- and 4-kinase family protein*
1.34	1.52	2.96	1.76	Roisl.0209s0122	AT1G33055	HUP32
-1.49	-0.74	1.71	3.79	Roisl.0048s0036	AT1G35140	PHI-1
-0.56	1.37	NA	0.13	Roisl.0048s0037	AT1G35140	PHI-1
0.94	2.29	5.8	4.05	Roisl.0185s0012	AT1G43800	FTM1
NA	NA	NA	NA	NA	AT1G55810	UCK3
-2.06	-1.77	1.43	1.12	Roisl.0010s0125	AT1G63090	PP2-A11
-0.35	-0.25	-0.75	-0.54	Roisl.0007s0399	AT1G72330	ALAAT2
0.59	0.21	-0.85	-1.24	Roisl.0019s0266	AT1G72330	ALAAT2
NA	NA	NA	NA	NA	AT1G72940	TN11
1.76	2.36	2.32	2.74	Roisl.0004s0404	AT1G74940	*putative cyclin-dependent kinase*
2.74	5.13	5.28	4.92	Roisl.0004s0224	AT1G76650	CML38
2.46	2.6	1.5	3.34	Roisl.0004s0185	AT1G77120	ADH1
0.38	0.32	0.31	0.13	Roisl.0045s0005	AT2G16060	HB1
0.57	0.95	0.34	1.79	Roisl.0045s0006	AT2G16060	HB1
0.13	0.18	0.45	2.33	Roisl.0066s0138	AT2G17850	*Rhodanese/Cell cycle control phosphatase superfamily protein*
1.47	1.41	0.12	0.11	Roisl.0214s0359	AT2G19590	ACO1
NA	NA	NA	NA	Roisl.0028s0844	AT2G34390	NIP2;1
1.6	0.41	1.03	0.84	Roisl.0067s0064	AT2G47520	HRE2
0.99	2.26	1.36	2.36	Roisl.0016s0187	AT3G02550	LBD41
-0.79	-0.78	-4.06	-0.3	Roisl.0210s1307	AT3G02550	LBD41
-0.34	-0.05	0.13	0.14	Roisl.0074s1020	AT3G10040	HRA1
0.14	-0.04	0.18	0.29	Roisl.0074s0150	AT3G17860	JAZ3
5.67	6.28	4.17	5.1	Roisl.0053s0381	AT3G23150	ETR2
0.13	0.25	0.4	0.57	Roisl.0053s0378	AT3G23170	PRP
-1.27	-0.27	-1.12	-1.24	Roisl.0011s0514	AT3G27220	HUP6
-0.31	-0.9	1.11	0.83	Roisl.0208s0037	AT3G43190	SUS4
NA	NA	0.07	0.77	Roisl.0046s0676	AT3G47720	SRO4
3.8	3.61	4.23	3.71	Roisl.0018s0219	AT3G61060	PP2-A13
NA	NA	NA	NA	Roisl.0020s0095	AT4G10265	WIP3
NA	3.84	1.44	1.4	Roisl.0040s0084	AT4G10265	WIP3
3.18	3.58	0.94	0.79	Roisl.0040s0091	AT4G10265	WIP3
NA	NA	3.87	5.03	Roisl.0020s0096	AT4G10270	WIP4
3.04	3.76	4.14	4.67	Roisl.0020s0097	AT4G10270	WIP4

-3 0 3 log_2FC

R. stylosa 24 h	*R. stylosa* 48 h	*R. islandica* 24 h	*R. islandica* 48 h	Rorippa-ID	AGI	Annotation
NA	NA	NA	NA	Roisl.0020s0099	AT4G10270	WIP4
2.88	2.43	2.03	NA	Roisl.0020s0101	AT4G10270	WIP4
2.61	4.15	3.65	2.53	Roisl.0020s0106	AT4G10270	WIP4
3.03	3.75	1.47	1.8	Roisl.0020s0108	AT4G10270	WIP4
1.82	3.9	3.83	7.44	Roisl.0040s0083	AT4G10270	WIP4
2.64	1.12	2.38	2.1	Roisl.0040s0085	AT4G10270	WIP4
2.25	4.36	-0.98	-1.42	Roisl.0040s0092	AT4G10270	WIP4
3.86	4.53	-0.28	0.8	Roisl.0040s0093	AT4G10270	WIP4
1.05	1.65	-0.92	-0.18	Roisl.0091s0562	AT4G17670	*senescence-associated family protein*
0.14	0.94	1.46	2.21	Roisl.0091s0041	AT4G22780	ACR7
2.87	3.61	1.05	2.78	Roisl.0008s0223	AT4G24110	HUP40
4.93	4.81	5.1	5.07	Roisl.0022s0406	AT4G27450	HUP54
-0.04	0.25	1.34	0.92	Roisl.0003s0317	AT4G32840	PFK6
-1.86	-2.21	-3.25	-1.22	Roisl.0003s0343	AT4G33070	PDC1
0.09	-0.49	-2.38	-2.5	Roisl.0008s0299	AT4G33560	WIP5
NA	NA	NA	NA	Roisl.0001s0502	AT4G39675	*hypothetical protein*
0.8	1.24	0.22	0.38	Roisl.0034s0126	AT5G02200	FHL
3.45	2.35	3.7	2.01	Roisl.0210s0718	AT5G10040	HUP9
NA	NA	2.61	1.93	Roisl.0210s0719	AT5G10040	HUP9
1.52	2.4	-1.07	-0.03	Roisl.0210s1214	AT5G15120	PCO1
0.91	0.81	1.26	0.74	Roisl.0046s0296	AT5G26200	*Mitochondrial substrate carrier family protein*
0.92	1.31	5.35	3.76	Roisl.0009s0276	AT5G39890	PCO2
-0.49	-0.38	0.3	0.62	Roisl.0075s0031	AT5G42200	ATL23
0.51	0.92	0.57	0.32	Roisl.0012s0334	AT5G44730	*Haloacid dehalogenase-like hydrolase superfamily protein*
3.1	4.55	0.88	2.83	Roisl.0005s0317	AT5G45340	CYP707A3
-0.77	0.07	-2.55	-1.89	Roisl.0005s0076	AT5G47060	*hypothetical protein*
NA	NA	NA	NA	Roisl.0046s0607	AT5G47060	*hypothetical protein*
0.27	0.38	0.38	0.79	Roisl.0206s0003	AT5G47910	RBOHD
0.3	0.95	0.11	0.11	Roisl.0080s0842	AT5G54960	PDC2
1.13	1.76	0.89	1.43	Roisl.0080s0487	AT5G58070	TIL
4.79	3.93	6.19	4.68	Roisl.0080s0110	AT5G61440	ACHT5
NA	NA	1	0.86	Roisl.0024s0457	AT5G61440	ACHT5
3.16	5.26	NA	NA	Roisl.0209s0225	AT5G61440	ACHT5
1.59	2.27	2.95	1.83	Roisl.0024s0119	AT5G62520	SRO5
-0.5	0.5	-2.64	-2.06	Roisl.0024s0569	AT5G66985	HUP44

Supplementary Figure S1: Heatmap of hypoxia-responsive genes. Transcripts with homology to previously defined HRGs (Mustroph et al. 2009, Renziehausen et al. 2025) were selected. Values are log2FC of submergence vs control for 24 and 48 h treatment in *R. stylosa* and *R. islandica*, as indicated by the scale. Transcripts with no significant changes (Padj > 0.05) are shown in grey. NA, no homologous gene found in the *R. islandica* genome, or no transcript detected.

R. stylosa 24 h	*R. stylosa* 48 h	*R. islandica* 24 h	*R. islandica* 48 h	Rorippa-ID	AGI	Annotation
2.39	2.67	5.33	4.17	Roisl.0210s0249	AT1G03090	MCCA
1.37	1.9	2.08	1.68	Roisl.0091s0925	AT1G06570	PDS1
0.56	0.8	3.07	2.16	Roisl.0091s1151	AT1G08630	THA1
6.41	7.03	8.19	6.78	Roisl.0021s0056	AT1G10070	BCAT-2
0.98	1.12	6.75	6.86	Roisl.0191s0240	AT1G10070	BCAT-2
2.13	2.23	2.65	2.18	Roisl.0021s0203	AT1G11260	STP1
1.61	1.37	1.67	1.25	Roisl.0021s0369	AT1G12780	UGE1
-0.14	-1.22	-0.01	-0.45	Roisl.0007s0631	AT1G15010	*mediator of RNA polymerase II transcription subunit*
3.05	4.81	1.09	2.36	Roisl.0007s0628	At1G15040	GAT1_2.1
5.11	6.63	4.32	3.25	Roisl.0007s0594	AT1G15380	GLY14
6.22	9.26	-0.14	-0.75	Roisl.0078s0013	AT1G15380	GLY14
1.47	1.82	0.91	0.94	Roisl.0007s0310	AT1G18270	*ketose-bisphosphate aldolase class-II family protein*
8.72	10.11	6.99	7.61	Roisl.0007s0177	AT1G19530	RGAT1
3.19	3.51	3.84	3.47	Roisl.0049s0047	AT1G21400	*Thiamin diphosphate-binding fold (THDP-binding) superfamily protein*
3.75	3.32	4.09	2.91	Roisl.0191s0220	AT1G23870	TPS9
5.25	5.94	6.03	6.39	Roisl.0213s0094	AT1G28330	DYL1
-1.49	-0.74	1.71	3.79	Roisl.0048s0036	AT1G35140	PHI-1
-0.76	1.37	NA	0.78	Roisl.0048s0037	AT1G35140	PHI-1
1.13	0.44	4.35	3.69	Roisl.0189s0051	AT1G54740	*FANTASTIC four-like protein* (DUF3049)
NA	NA	NA	NA	NA	AT1G55810	UKL3
1.86	2.14	4.68	4.34	Roisl.0214s0623	AT1G60140	TPS10
0.11	0.15	-0.11	0.49	Roisl.0214s0622	AT1G60160	*Potassium transporter family protein*
-0.69	-0.6	-2.81	-3.31	Roisl.0209s0547	AT1G62510	*Hydrolase superfamily protein located in chloroplast*
1.44	1.54	2.48	1.87	Roisl.0010s0137	AT1G63180	UEG3
1.44	2.02	1.1	1.2	Roisl.0050s0010	AT1G64660	MGL
3.71	3.24	3.52	3.52	Roisl.0019s0505	AT1G70290	TPS8
3.72	4.69	6.83	7.06	Roisl.0019s0422	AT1G71030	MYBL2
1.16	0.62	1.01	0.46	Roisl.0004s0249	AT1G76410	ATL8
2.49	3.06	3.35	4.13	Roisl.0004s0231	AT1G76590	*PLATZ transcription factor family protein*
1.46	1.92	2.03	5.44	Roisl.0004s0177	AT1G77210	STP14
2.9	2.99	5.17	4.12	Roisl.0115s0150	AT1G79700	WRI4
4.51	5.16	2	5.24	Roisl.0115s0053	AT1G80160	GLY17
5.63	5.11	8.09	8.17	Roisl.0115s0079	AT1G80440	KMD1
2.11	2.21	2.33	1.97	Roisl.0011s0160	AT2G02710	PLPB
-0.64	3.04	3.62	4.12	Roisl.0213s0694	AT2G05540	*Glycine-rich protein family*
3.53	4.56	6.09	7.37	Roisl.0213s0695	AT2G05540	*Glycine-rich protein family*
5.52	5.83	7.37	6.65	Roisl.0045s0019	AT2G15890	MEE14
3.53	3.54	7.45	6.81	Roisl.0045s0013	AT2G15960	*stress-induced protein*
5.27	4.78	6.8	6	Roisl.0066s0030	AT2G18700	TPS11
NA	NA	2.65	2.1	Roisl.0044s0070	AT2G19800	MIOX2
4.94	4.84	7.06	5.85	Roisl.0214s0383	AT2G19800	MIOX2
3.43	3.21	6.92	5.98	Roisl.0214s0490	AT2G20670	*sugar phosphate exchanger, putative* (DUF560)
NA	NA	NA	NA	NA	AT2G22980	SCPL13
4.48	4.15	4.47	3.37	Roisl.0118s0009	AT2G25900	ATCTH
1.07	1.99	2.83	2.34	Roisl.0033s0083	AT2G30600	*BTB/POZ domain-containing protein*
1.69	3.03	3.73	4.06	Roisl.0028s0789	AT2G33830	DRM2
3.41	3.91	3.44	2.71	Roisl.0017s0201	AT2G38400	AGT3
9.33	8.79	8.33	8.75	Roisl.0017s0313	AT2G39400	MAGL6
3.66	3.5	7.75	6.71	Roisl.0017s0386	AT2G40000	HSPRO2
0.77	0.9	0.92	0.92	Roisl.0028s0268	AT2G43400	ETFQO

R. stylosa 24 h	*R. stylosa* 48 h	*R. islandica* 24 h	*R. islandica* 48 h	Rorippa-ID	AGI	Annotation
1.36	1.61	2.85	1.82	Roisl.0079s0073	AT3G06850	BCE2
2.89	2.72	4.7	4.84	Roisl.0079s0128	AT3G07350	*sulfate/thiosulfate import ATP-binding protein, putative* (DUF506)
2.31	2.34	2.43	2.72	Roisl.0074s1022	AT3G10020	*plant/protein*
1.5	1.32	3.02	2.45	Roisl.0074s0645	AT3G13450	DIN4
7.03	6.7	8.47	6.3	Roisl.0006s0360	AT3G15450	*aluminium induced protein with YGL and LRDR motifs*
5.68	4.96	8.08	6.06	Roisl.0074s0411	AT3G15450	*aluminium induced protein with YGL and LRDR motifs*
4.49	4	7.5	5.44	Roisl.0074s0386	AT3G15630	*plant/protein*
3.98	5.73	7.12	9.94	Roisl.0115s0356	AT3G20340	*protein expression protein*
NA	NA	NA	NA	NA	AG3G20660	OCT4
5.89	5.33	7.3	4.68	Roisl.0185s0084	AT3G30775	ERD5
2.84	3.06	3.28	2.46	Roisl.0020s0158	AT3G45300	IVD
1.94	2.49	7.68	6.55	Roisl.0209s0438	AT3G47340	ASN1
3.78	3.56	6.74	5.66	Roisl.0026s0141	AT3G57520	SIP2
3.8	3.61	4.23	3.71	Roisl.0018s0219	AT3G61060	PP2-A13
2.51	3.75	1.53	0.78	Roisl.0018s0375	AT3G62550	*Adenine nucleotide alpha hydrolases-like superfamily protein*
3.22	3.63	3.74	3.88	Roisl.0018s0389	AT3G62550	*Adenine nucleotide alpha hydrolases-like superfamily protein*
3.23	1.7	3.77	2.4	Roisl.0018s0433	AT3G62950	*Thioredoxin superfamily protein*
2.92	2.82	0.94	0.64	Roisl.0191s0609	AT4G01870	*tolB protein- like protein*
0.81	1.36	2	1.75	Roisl.0082s0320	AT4G03510	RMA1
3.76	3.54	6.43	5.82	Roisl.0022s0285	AT4G26260	MIOX4
4.93	4.81	5.1	5.07	Roisl.0022s0406	AT4G27450	*aluminium induced protein with YGL and LRDR motifs*
2.52	3.28	4.26	4.67	Roisl.0003s0036	AT4G30270	XTH24
2.14	1.82	3.37	2.27	Roisl.0008s0356	AT4G34030	MCCB
0.66	1.01	1.3	1.31	Roisl.0001s0001	AT4G34138	UGT73B1
0.35	0.97	1.14	1.33	Roisl.0001s0005	AT4G34138	UGT73B1
6.76	7.9	8.24	8.17	Roisl.0001s0198	AT4G35770	SEN1
3.48	3.41	4.18	3.53	Roisl.0001s0229	AT4G36040	J11
5.97	6.27	7.14	6.83	Roisl.0001s0287	AT4G36670	PMT6
2.93	2.63	4.21	3.24	Roisl.0115s0750	AT4G38470	STY46
1.27	2.2	1.13	2.6	Roisl.0034s0046	AT5G01600	FER1
1.92	2.1	1.24	3.75	Roisl.0079s0245	AT5G01600	FER1
0.27	0.77	-0.06	0.14	Roisl.0034s0166	AT5G02810	PRR7
-0.11	-0.46	2.41	1.29	Roisl.0210s0483	AT5G07440	GDH2
4.14	3.76	2.87	1.7	Roisl.0210s0581	AT5G08350	*GRAM domain-containing protein / ABA-respnsive protein-like protein*
0.23	0.5	-0.36	-0.34	Roisl.0210s1388	AT5G16840	*AMP-dependent synthetase and ligase family protein*
-1.14	-1.11	1.57	1.19	Roisl.0210s1525	AT5G18170	GDH1
4.18	3.74	6.62	5.33	Roisl.0210s1631	AT5G19120	*Eukaryotic aspartyl protease family protein*
4.44	5.68	2.85	3.81	Roisl.0210s1751	AT5G20230	BCB
4.54	4.69	5.99	4.71	Roisl.0210s1753	AT5G20250	DIN10
2.28	2.31	4.26	3.78	Roisl.0210s1844	AT5G21170	AKINBETA1
2.6	3.01	4.46	2.98	Roisl.0210s1954	AT5G22920	*CHY-type/CTCHY-type/RING-type Zinc finger protein*
2.82	3.22	5.65	4.8	Roisl.0036s0090	AT5G49360	BXL1
3.73	3.3	4.15	3.55	Roisl.0036s0084	AT5G49450	bZIP1
0.47	0.23	1.4	1.04	Roisl.0189s0237	AT5G51390	*hypothecial protein*
2.07	2.63	0.13	0.78	Roisl.0022s0308	AT5G56100	*glycine-rich protein / oleosin*
NA	1.62	4.75	2.37	Roisl.0080s0719	AT5G56100	*glycine-rich protein / oleosin*
5.7	6.11	8.3	7.78	Roisl.0080s0622	AT5G56870	BGAL4
1.49	2.42	0.93	2.02	Roisl.0080s0545	AT5G57560	TCH4
1.34	1.92	1.16	1.58	Roisl.0080s0535	AT5G57655	*xylose isomerase family protein*

Scale: -3 0 3 log_2FC

Supplementary Figure S2: Heatmap of carbon starvation-responsive genes. Transcripts with homology to carbon starvation markers (Usadel et al. 2008, Cookson et al. 2016) were selected. Values are log2FC of submergence vs control for 24 and 48 h treatment in *R. stylosa* and *R. islandica*, as indicated by the scale. Transcripts with no significant changes (Padj > 0.05) are shown in grey. NA, no homologous gene found in the *R. islandica* genome, or no transcript detected.

Scale: -3 0 3 log_2FC

R. stylosa 24 h	R. stylosa 48 h	R. islandica 24 h	R. islandica 48 h	Rorippa-ID	AGI	Annotation
2.13	2.4	3.15	2.33	Roisl.0210s0366	AT1G04310	ERS2
4.15	3.92	2.19	2.24	Roisl.0091s0885	AT1G06160	ORA59
0.6	1.03	1.3	1.21	Roisl.0021s0211	AT1G11330	RDA2
0.02	1.12	-0.83	-0.32	Roisl.0021s0280	AT1G11920	PLL6
0.02	0.42	-1.01	-0.99	Roisl.0007s0643	AT1G14890	*Plant invertase/pectin methylesterase inhibitor superfamily protein*
8.72	10.11	6.99	7.61	Roisl.0007s0177	AT1G19530	RGAT1
5.18	7.76	0.23	0.24	Roisl.0049s0039	AT1G21320	NSRB
NA	NA	NA	NA	NA	AT1G33790	*jacalin lectin family protein*
0.98	1.12	NA	NA	Roisl.0006s0020	AT1G49570	IRR1
3.17	4.79	3.09	3.46	Roisl.0006s0234	AT1G51790	*Leucine-rich repeat protein kinase family protein*
4.71	5.03	4.95	4.2	Roisl.0019s0263	AT1G72360	HRE1
1.08	1.28	0.18	0.2	Roisl.0004s0355	AT1G75390	bZIP44
1.16	0.62	1.01	0.46	Roisl.0004s0249	AT1G76410	ATL8
2.04	2.65	4.37	4.11	Roisl.0004s0160	AT1G77380	AAP3
1.51	1.69	2.31	2.26	Roisl.0213s0744	AT2G04795	*hypothetical protein*
1.47	1.41	0.12	0.11	Roisl.0214s0359	AT2G19590	ACO1
NA	NA	NA	NA	NA	AT2G23590	MES8
0.46	0.79	0.5	0.26	Roisl.0082s0504	AT2G26070	RTE1
9.33	8.79	8.33	8.45	Roisl.0017s0313	AT2G39400	MAGL6
2.45	2.17	5.67	3.68	Roisl.0017s0384	AT2G39980	*HXXXD-type acyl-transferase family protein*
3.08	3.19	2.42	2.05	Roisl.0067s0065	AT2G40940	ERS1
-0.24	0.67	-0.84	-0.37	Roisl.0028s0526	AT2G40970	MYBC1
NA	NA	NA	NA	NA	AT2G41100	CAL4
NA	NA	NA	NA	NA	AT2G41230	ARL2
-2.69	-2.28	0.66	2.07	Roisl.0028s0330	AT2G42850	CYP718
7.42	8.04	6.76	6.92	Roisl.0028s0297	AT2G43140	BHLH129
5.17	5.5	4.78	4.39	Roisl.0028s0182	AT2G44080	ARL
-1.16	-1.75	-1.41	-1.68	Roisl.0035s0022	AT2G47160	BOR1
2.03	0.42	0.03	-1.05	Roisl.0067s0061	AT2G47550	PMEI-PME20
0.47	0.67	-1.2	-1.14	Roisl.0067s0007	AT2G48090	*hypothetical protein*
NA	NA	NA	NA	NA	AT3G01730	*Mutants exhibit shorter root hairs under phosphate-deficient conditions*
-1.69	-1.51	0.81	0.43	Roisl.0074s0886	AT3G11210	GGL17
-1.24	0.07	1.08	0.81	Roisl.0074s0420	AT3G15370	EXP12
3.57	3.72	0.61	0.84	Roisl.0115s0500	AT3G21510	AHP1
0.52	0.82	2.45	3.01	Roisl.0115s0623	AT3G22750	RAF39
2.14	2.09	1.05	0.87	Roisl.0053s0399	AT3G23030	IAA2
5.67	6.28	4.17	5.1	Roisl.0053s0381	AT3G23150	ETR2
-0.63	-1.39	1.74	1.11	Roisl.0011s0335	AT3G25730	EDF2
3.54	3.82	3.53	3.25	Roisl.0046s0783	AT3G48580	XTH11
8.96	9.16	7.88	6.05	Roisl.0046s1091	AT3G51410	*hypothetical protein*

Scale: -3 0 3 log_2FC

R. stylosa 24 h	R. stylosa 48 h	R. islandica 24 h	R. islandica 48 h	Rorippa-ID	AGI	Annotation
-1.68	-1.66	-0.49	-0.25	Roisl.0046s1203	AT3G52340	SPP2
4.14	4.61	5.02	4.26	Roisl.0018s0101	AT3G59900	ARGOS
-2.05	-2.26	-0.29	-1.39	Roisl.0018s0175	AT3G60550	CYCP3;2
1.41	1.12	3.8	2.9	Roisl.0082s0353	AT4G03140	*NAD(P)-binding Rossmann-fold superfamily protein*
-0.26	-0.04	0.09	1.94	Roisl.0020s0011	AT4G07960	CSLC12
NA	NA	-0.83	NA	Roisl.0015s0188	AT4G21400	CRK28
2.75	3.49	7.3	7.2	Roisl.0091s0190	AT4G21410	CRK29
3.45	4.39	1.51	1.81	Roisl.0091s0191	AT4G21410	CRK29
2.85	3.8	0.59	-0.55	Roisl.0091s0192	AT4G21410	CRK29
4.8	8.95	4.3	4.4	Roisl.0091s0193	AT4G21410	CRK29
2.06	2.19	1.07	0.58	Roisl.0091s0137	AT4G21870	*HSP20-like chaperone*
-0.11	1.71	NA	NA	Roisl.0022s0230	AT4G25790	CAP24
0.72	1.21	-3.33	-2.57	Roisl.0115s0755	AT4G38410	*Dehydrin family protein*
0.87	0.36	-1.21	-1.89	Roisl.0034s0088	AT5G01210	*HXXXD-type acyl-transferase family protein*
0.37	0.2	0.34	-0.71	Roisl.0034s0159	AT5G02550	hypothetical protein
4.34	4.36	-0.59	-2.89	Roisl.0034s0171	AT5G02760	APD7
0.71	1.26	-0.48	-0.94	Roisl.0042s0005	AT5G06570	CXE15
0.98	0.5	-1.34	-3.57	Roisl.0210s1554	AT5G18470	*Curculin-like (mannose-binding) lectin family protein*
1.05	0.79	-0.45	-0.85	Roisl.0210s1555	AT5G18470	*Curculin-like (mannose-binding) lectin family protein*
2.13	NA	NA	NA	Roisl.0210s1712	AT5G19890	*Peroxidase superfamily protein*
2.31	0.11	-0.14	0.18	Roisl.0210s1790	AT5G20640	*LURP-one-like protein*
-2.51	-1.87	NA	NA	Roisl.0210s1805	AT5G20820	SAUR76
-3.12	-2.52	-6.93	-6.28	Roisl.0046s0197	AT5G25190	ESE3
2.19	2.82	2.18	2.33	Roisl.0046s0218	AT5G25350	EBF2
5.57	5.1	6.7	5.37	Roisl.0046s0485	AT5G28770	bZIP63
NA	NA	NA	NA	NA	AT5G38930	DEG8
6.86	7.82	7.25	8.05	Roisl.0009s0317	AT5G39610	ANAC092
1.82	1.12	-2.66	0.52	Roisl.0009s0174	AT5G40590	*Cysteine/Histidine-rich C1 domain family protein*
4.18	5.11	2.05	3.09	Roisl.0009s0152	AT5G40780	LHT1
NA	NA	NA	NA	Roisl.0011s0520	AT5G40780	LHT1
NA	NA	NA	NA	NA	AT5G48175	*transmembrane protein*
1.5	1.38	-0.87	NA	Roisl.0052s0065	AT5G53980	HB52
0.26	1.87	-0.35	-2.05	Roisl.0184s0123	AT5G53980	HB52
-2.9	-5.2	0.14	-1.04	Roisl.0080s0504	AT5G57920	ENODL10
4.59	5.72	2.02	0.37	Roisl.0080s0018	AT5G62280	*DUF1442 family protein*
NA	NA	NA	-1.15	Roisl.0024s0245	AT5G63660	LCR74
NA	NA	NA	NA	Roisl.0024s0109	AT5G64110	*Peroxidase superfamily protein*
3.61	5.57	2.79	3.19	Roisl.0024s0291	AT5G64110	*Peroxidase superfamily protein*
-1.02	-0.94	-1.2	-1.43	Roisl.0024s0508	AT5G67520	APK4

Supplementary Figure S3: Heatmap of ethylene-responsive genes. Transcripts with homology to ethylene-responsive transcripts (Goda et al. 2008, Harkey et al. 2018, Liu et al. 2022) were selected. Values are log2FC of submergence vs control for 24 and 48 h treatment in *R. stylosa* and *R. islandica*, as indicated by the scale. Transcripts with no significant changes (Padj > 0.05) are shown in grey. NA, no homologous gene found in the *R. islandica* genome, or no transcript detected.

A

R. stylosa 24 h	R. stylosa 48 h	R. islandica 24 h	R. islandica 48 h	Rorippa-ID	AGI	Annotation	Function
NA	NA	-1.84	NA	Roisl.0028s0621	AT1G05230	HDG2	Differentiation
-0.75	-0.76	-0.91	-0.82	Roisl.0091s0774	AT1G05230	HDG2	
0.6	0.3	-1.11	-1.42	Roisl.0021s0379	AT1G12860	SCRM2	
-0.99	-1.05	-2.8	-1.5	Roisl.0007s0713	AT1G14350	FLP	
0.01	0.16	0.4	0.63	Roisl.0011s0174	AT2G02820	MYB88	
0.54	0.28	-2.42	-3.11	Roisl.0039s0159	AT3G06120	MUTE	
0.6	0.97	0.17	0.21	Roisl.0053s0276	AT3G24140	FMA	
0.07	-0.07	-0.04	-0.14	Roisl.0011s0454	AT3G26744	ICE1	
-0.3	-0.91	-1.11	-1.42	Roisl.0031s0096	AT5G53210	SPCH	
-1.31	-1.47	-3.57	-3.95	Roisl.0210s0346	AT1G04110	SDD1	Spacing and pattering
1.83	0.76	1.92	2.6	Roisl.0007s0301	AT1G18350	MKK7	
-0.05	-1.59	-1.94	-1.34	Roisl.0056s0028	AT1G34245	EPF2	
-0.05	0.46	0.06	0.19	Roisl.0006s0222	AT1G51660	MKK4	
0.43	0.79	0.26	0.38	Roisl.0010s0194	AT1G63700	YDA	
2.99	3.3	3.2	2.48	Roisl.0019s0133	AT1G73500	MKK9	
NA	NA	NA	NA	NA	AT1G80080	TMM	
0.41	0.34	-2.13	-3.2	Roisl.0214s0522	AT2G20875	EPF1	
-1.36	-1.77	-3.16	-3.78	Roisl.0198s0059	AT2G26330	ER	
0.98	1.24	-5.52	-4.25	Roisl.0033s0055	AT2G30370	CHAL	
-0.46	-0.01	-1.35	-2.09	Roisl.0017s0411	AT2G40180	PP2C5	
0.1	0.18	0.1	0.11	Roisl.0028s0214	AT2G43790	MPK6	
-1.02	-1.05	-0.93	-0.85	Roisl.0115s0464	AT3G21220	MKK5	
1.79	2.43	1.64	1.73	Roisl.0209s0272	AT3G45640	MPK3	
0.23	0.12	0.06	-0.35	Roisl.0082s0019	AT4G12970	STOMAGEN	
-0.77	-0.93	-1.67	-1.72	Roisl.0210s0454	AT5G07180	ERL2	
-0.09	-0.38	-1.36	-1.43	Roisl.0080s0023	AT5G62230	ERL1	
0.09	-0.14	-1.23	-2.13	Roisl.0028s0392	AT2G42290	PNL2	Polarity and Division Asymmetry
-0.85	-1.3	-0.87	-2.83	Roisl.0026s0105	AT3G57830	PNL1	
-0.92	-1.52	-2.58	-2.39	Roisl.0003s0196	AT4G31805	POLAR	
-0.89	-1.19	-1.93	-2.12	Roisl.0080s0181	AT5G60880	BASL	
-0.52	-1.12	-2.51	-3.69	Roisl.0007s0573	AT1G15570	CYCA2;3	Cell division
-0.3	-0.25	-0.17	0.03	Roisl.0013s0046	AT1G49040	SCD1	
-0.89	-1.43	-1.22	-2.36	Roisl.0115s0073	AT1G80370	CYCA2;4	
0.06	0.31	0.27	0.5	Roisl.0017s0019	AT2G36850	GSL8	
-0.04	0.37	0.15	0.41	Roisl.0032s0060	AT2G36850	GSL8	
NA	NA	-3.04	-4.23	Roisl.0017s0227	AT2G38620	CDKB1;2	
-1.16	-2.24	-0.66	-2.27	Roisl.0046s1396	AT3G54180	CDKB1;1	
-0.52	-0.72	-0.67	-1.62	Roisl.0210s0761	AT5G10440	CYCD4;2	
-2.28	-2.49	-1.47	-0.68	Roisl.0210s0846	AT5G11300	CYCA2;2	
-2.49	-5.69	-2.05	-2.6	Roisl.0046s0213	AT5G25380	CYCA2;1	

R. stylosa 24 h	R. stylosa 48 h	R. islandica 24 h	R. islandica 48 h	Rorippa-ID	AGI	Annotation	Function
0.02	-1.74	-3.39	-2.27	Roisl.0210s0281	AT1G03445	BSU1	Hormone and Environmental Signaling
2.15	2.13	1.77	1.74	Roisl.0210s0375	AT1G04400	CRY2	
1.98	2.21	1.83	1.96	Roisl.0210s0376	AT1G04400	CRY2	
NA	NA	NA	NA	Roisl.0012s0101	AT1G04400	CRY2	
1.49	1.63	1.13	1.47	Roisl.0091s1239	AT1G09570	PHYA	
-0.52	-0.24	-1.98	-1.58	Roisl.0074s0238	AT2G18790	PHYB	
0.39	0.51	0.87	1.06	Roisl.0028s0683	AT2G32950	COP1	
-0.68	-1.04	-0.51	-0.51	Roisl.0028s0313	AT2G43010	PIF4	
1.67	1.65	3.72	2.57	Roisl.0035s0067	AT2G46720	KCS13	
1.38	1.54	1.68	1.41	Roisl.0074s0634	AT3G13550	FUS9	
1.04	0.97	0.91	0.89	Roisl.0032s0062	AT4G08920	CRY1	
1.46	1.39	0.69	0.59	Roisl.0091s0440	AT4G18710	BIN2	
-2.02	-1.87	-1.13	-2.94	Roisl.0115s0019	AT1G79840	GL2	Hypocotyl Stomatal Genes
-3.68	-4.07	-10.7	-10.6	Roisl.0016s0067	AT5G14750	MYB66	
0.25	0.45	0.5	0.5	Roisl.0046s0132	AT5G24520	TTG1	
-0.34	0.24	-0.87	-0.45	Roisl.0210s0112	AT1G01260	JAM2	added from Chowdhury et al. 2021
1.64	1.78	1.79	1.73	Roisl.0007s0591	AT1G15400	MASS2	
0.73	0.92	0.84	0.75	Roisl.0016s0114	AT3G01090	KIN10	
0.31	0.31	0.26	0.31	Roisl.0024s0453	AT5G65590	SCAP1	

Scale: -3 0 3 log_2FC

B

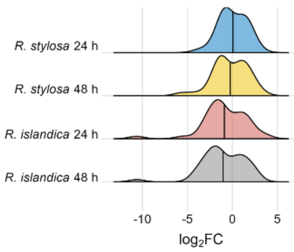

Supplementary Figure S4: Heatmap of transcripts involved in stomata formation. Transcripts with homology to proteins with a known function in stomata biogenesis (Pillitteri and Dong 2013, Chowdhury et al. 2021) were selected. (A) Values are log2FC of submergence vs control for 24 and 48 h treatment in *R. stylosa* and *R. islandica*, as indicated by the scale. Transcripts with no significant changes (Padj > 0.05) are shown in grey. NA, no homologous gene found in the *R. islandica* genome, or no transcript detected. (B) Ridgeline plot of DEGs presented in (A). The height of the plot represents the number of DEGs with the respective log2FC. The mean is marked with a black line.

A

R. stylosa 24 h	*R. stylosa* 48 h	*R. islandica* 24 h	*R. islandica* 48 h	Rorippa-ID	AGI	Annotation	Protein localization
-0.79	-0.89	-3.01	-4.3	Roisl.0016s0066	AT3G01500	BCA1	Chloroplast
0.12	-0.42	2.1	1.28	Roisl.0210s1176	AT5G14740	BCA2	Cytosol
1.23	2.06	6.73	7.86	Roisl.0191s0207	AT1G23730	BCA3	Cytosol
0.08	-0.15	-0.18	-0.54	Roisl.0019s0496	AT1G70410	BCA4	Cytosol / Plasma membrane
-0.04	0.04	-0.24	-0.27	Roisl.0008s0301	AT4G33580	BCA5	Chloroplast
3.49	3.59	5.59	4.95	Roisl.0214s0763	AT1G58180	BCA6	Mitochondrion

-3 0 3
log_2FC

B

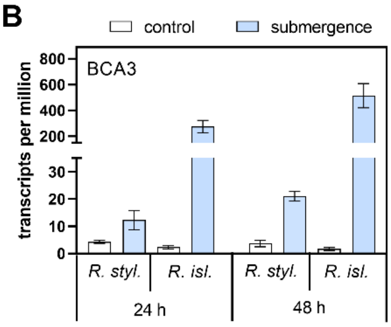

C

R. stylosa 24 h	*R. stylosa* 48 h	*R. islandica* 24 h	*R. islandica* 48 h	Rorippa-ID	AGI	Annotation
-2.67	-3.88	-4.61	-6.5	Roisl.0046s1239	AT3G52720	ACA1
NA	NA	NA	NA	NA	AT2G28210	ACA2
-0.84	1.75	-0.87	0.78	Roisl.0030s0146	AT5G04180	ACA3
NA	NA	-3.36	-2.14	Roisl.0091s0233	AT4G20990	ACA4
NA	NA	-1.16	0.13	Roisl.0091s0236	AT4G20990	ACA4
1.01	-1.03	0.45	-0.98	Roisl.0091s1087	AT1G08065	ACA5
NA	NA	NA	NA	NA	AT4G21000	ACA6
1.18	0.16	NA	NA	Roisl.0082s0571	AT1G08080	ACA7
NA	NA	3.62	-0.18	Roisl.0091s1089	AT1G08080	ACA7
NA	NA	NA	NA	NA	AT5G56330	ACA8
-0.68	-0.94	-0.43	-0.62	Roisl.0007s0174	AT1G19580	GCA1
-0.65	-1.12	-0.75	-0.79	Roisl.0066s0332	AT1G47260	GCA2
-0.37	-0.65	-0.79	-1.05	Roisl.0024s0629	AT5G66510	GCA3
-0.5	-0.86	-0.43	-1.05	Roisl.0024s0228	AT5G63510	GCAL1
-0.4	-0.55	-0.28	-0.38	Roisl.0046s0789	AT3G48680	GCAL2

-3 0 3
log_2FC

Supplementary Figure S5: Expression of Carbonic anhydrases in *Rorippa* species. (A) beta Carbonic anhydrases. Values are log2FC of submergence vs control for 24 and 48 h treatment in *R. stylosa* and *R. islandica*, as indicated by the scale. Transcripts with no significant changes (Padj > 0.05) are shown in grey. (B) Absolute transcript levels of *BCA3* transcripts shown in (A). Data are means +/- SD from three independent replicates. (C) alpha and gamma Carbonic anhydrases. Values are log2FC of submergence vs control for 24 and 48 h treatment in *R. stylosa* and *R. islandica*, as indicated by the scale. Transcripts with no significant changes (Padj > 0.05) are shown in grey. NA, no homologous gene found in the *R. islandica* genome, or no transcript detected.

-3 0 3 $\log_2$FC

R. stylosa 24 h	*R. stylosa* 48 h	*R. islandica* 24 h	*R. islandica* 48 h	Rorippa-ID	AGI	Annotation	*R. stylosa* 24 h	*R. stylosa* 48 h	*R. islandica* 24 h	*R. islandica* 48 h	Rorippa-ID	AGI	Annotation
-0.92	-1.18	-0.19	-0.13	Roisl.0210s0377	AT1G04410	NDH1	-0.93	-1.29	-1.98	-1.87	Roisl.0209s0459	AT3G47520	MDH
0.46	-0.73	-1.63	-2.16	Roisl.0091s0819	AT1G05610	APS2	-0.43	-0.38	-0.24	-0.25	Roisl.0191s0493	AT4G00570	NAD-ME2
1.49	1.63	1.13	1.47	Roisl.0091s1239	AT1G09570	FHY2	2.3	2.51	2.24	1.9	Roisl.0002s0148	AT4G15530	PPDK
NA	NA	NA	NA	NA	AT1G17290	AlaAT1	NA	NA	NA	NA	NA	AT4G16250	PHYD
-0.51	-0.49	-0.2	-0.46	Roisl.0191s0170	AT1G23310	AOAT1	0.05	0.2	-0.54	-0.26	Roisl.0091s0513	AT4G18130	PHYE
1.23	2.06	6.73	7.86	Roisl.0191s0207	AT1G23730	BCA3	-0.57	0.42	-0.38	0.23	Roisl.0022s0103	AT4G24570	DIC2
-0.55	-0.37	-1.31	-1.35	Roisl.0213s0031	AT1G27680	APL2	-0.29	-0.46	-0.86	-0.77	Roisl.0003s0214	AT4G31990	AAT3
-0.23	-0.24	NA	NA	Roisl.0006s0045	AT1G49810	NHD2	1.63	1.8	0.29	-0.06	Roisl.0001s0417	AT4G37870	PCK1
-1.18	-1.56	-1.44	-1.54	Roisl.0006s0395	AT1G53240	mMDH1	-2.19	-1.78	-1.96	-1.06	Roisl.0115s0675	AT4G39210	APL3
0.81	0.6	0.22	0.77	Roisl.0006s0403	AT1G53310	PEPC1	NA	NA	NA	NA	Roisl.0188s0109	AT4G39210	APL3
-1.64	-2.05	-2.61	-2.9	Roisl.0010s0077	AT1G62800	ASP4	2.92	3.75	-0.65	-0.85	Roisl.0210s0655	AT5G09470	DIC3
-0.64	-0.79	-0.66	-0.88	Roisl.0024s0094	AT1G67090	RBCS1A	-0.35	-0.38	0.09	-0.25	Roisl.0210s0678	AT5G09660	PMDH2
-0.94	-0.8	-2.14	-3.48	Roisl.0019s0678	AT1G68750	PPC4	1.63	2	0.69	0.97	Roisl.0210s0869	AT5G11520	ASP3
1.77	2.73	-1.7	-0.98	Roisl.0191s0403	AT1G68750	PPC4	1.39	2.2	0.54	1.05	Roisl.0210s0887	AT5G11670	NADP-ME2
0.08	-0.15	-0.18	-0.54	Roisl.0019s0496	AT1G70410	BCA4	-1.98	-2.48	-2.48	-3.03	Roisl.0210s0977	AT5G12860	DiT1
-0.82	-1.03	-0.87	-0.95	Roisl.0019s0475	AT1G70580	AOAT2	0.12	-0.42	2.1	1.28	Roisl.0210s1176	AT5G14740	BCA2
-1.81	1.12	0.01	-0.06	Roisl.0024s0606	AT1G70580	AOAT2	-0.38	-0.26	-0.68	-1.02	Roisl.0210s1641	AT5G19220	ADG2
-0.35	-0.25	-0.75	-0.54	Roisl.0007s0399	AT1G72330	ALAAT2	0.12	0	-0.97	-0.82	Roisl.0210s1674	AT5G19550	AAT2
0.59	0.21	-0.85	-1.24	Roisl.0019s0266	AT1G72330	ALAAT2	-0.85	-1.31	-0.99	-1.34	Roisl.0210s1696	AT5G19760	*novel mitochondrial carrier capable of transporting both dicarboxylates and tricarboxylates*
-0.69	-0.75	-1.22	-1.18	Roisl.0004s0040	AT1G78560	BASS1	0.99	-0.07	-4.72	-4.81	Roisl.0046s0268	AT5G25880	NADP-ME3
0.3	0.39	0.64	0.54	Roisl.0115s0017	AT1G79750	NADP-ME4	-0.1	-0.05	0.17	0.02	Roisl.0213s0017	AT5G33320	ARAPPT
0.21	0.29	0.91	0.93	Roisl.0066s0423	AT2G13560	NAD-ME1	NA	NA	-0.9	-0.04	Roisl.0214s0356	AT5G33320	ARAPPT
NA	NA	0.13	0.14	Roisl.0191s0480	AT2G13560	NAD-ME1	0.23	0.04	-1.15	-1.42	Roisl.0015s0154	AT5G35170	*adenylate kinase family protein*
0.98	NA	-0.12	-1.16	Roisl.0206s0036	AT2G13560	NAD-ME1	-0.09	-0.09	0	-0.08	Roisl.0053s0081	AT5G35840	PHYC
-0.52	-0.24	-1.98	-1.58	Roisl.0074s0238	AT2G18790	HY3	NA	NA	NA	NA	NA	AT5G38410	RBCS3B
-0.37	-0.51	0.08	0.38	Roisl.0214s0389	AT2G19900	NADP-ME1	NA	NA	NA	NA	NA	AT5G38420	RBCS2B
-1.04	-0.69	-1.33	-1.15	Roisl.0082s0121	AT2G21590	APL4	-1.36	-1.75	-2	-2.78	Roisl.0046s0003	AT5G38430	RBCS1B
-0.8	-1.08	-0.32	0.36	Roisl.0046s0588	AT2G22500	PUMP5	-0.36	-0.37	-0.32	-0.39	Roisl.0012s0096	AT5G43330	c-NAD-MDH2
0.32	0.25	0.34	0.34	Roisl.0046s0556	AT2G22780	PMDH1	-0.09	0.3	-0.68	-0.43	Roisl.0005s0025	AT5G47560	SDAT
-0.02	-0.64	-0.98	-0.96	Roisl.0029s0004	AT2G26900	BASS2	-0.24	0.24	-0.3	-0.18	Roisl.0011s0570	AT5G47840	AMK2
-1.81	-1.87	-1.43	-1.14	Roisl.0033s0154	AT2G30970	ASP1	-0.13	-0.15	-0.32	-0.72	Roisl.0188s0130	AT5G47840	AMK2
-1.45	-1.5	-0.02	0.06	Roisl.0028s0354	AT2G42600	PPC2	-0.06	0.32	-1.04	0.61	Roisl.0014s0025	AT5G48300	ADG1
-0.8	-0.89	-3.01	-4.3	Roisl.0016s0066	AT3G01500	BCA1	-1.27	-1.02	-1.96	-1.43	Roisl.0053s0430	AT5G48300	ADG1
-1.18	-1.79	-1	-1.96	Roisl.0016s0057	AT3G01550	PPT2	0.03	-0.78	-1.03	-2.38	Roisl.0080s0642	AT5G56720	c-NAD-MDH3
NA	NA	-0.02	-0.37	Roisl.0184s0081	AT3G01550	PPT2	-0.07	-0.1	-0.47	-0.56	Roisl.0080s0462	AT5G58330	NADP-MDH
-0.65	-0.44	-0.52	-0.24	Roisl.0074s0473	AT3G14940	PPC3	-0.69	-0.54	-3.37	-3.06	Roisl.0024s0308	AT5G64280	DiT2.2
-2.31	-2.7	-1.57	-2.88	Roisl.0074s0464	AT3G15020	mMDH2	-0.81	-1.03	-0.93	-1.12	Roisl.0024s0309	AT5G64290	DCT
-0.29	-0.21	-0.37	-0.53	Roisl.0028s0015	AT3G19490	NHD1	0.02	NA	5.8	6.02	Roisl.0024s0464	AT5G65690	PCK2

Supplementary Figure S6: Heatmap of transcripts with putative function in C4 and CAM photosynthesis (Goolsby et al. 2018). Values are log2FC of submergence vs control for 24 and 48 h treatment in *R. stylosa* and *R. islandica*, as indicated by the scale. Transcripts with no significant changes (Padj > 0.05) are shown in grey. NA, no homologous gene found in the *R. islandica* genome, or no transcript detected.

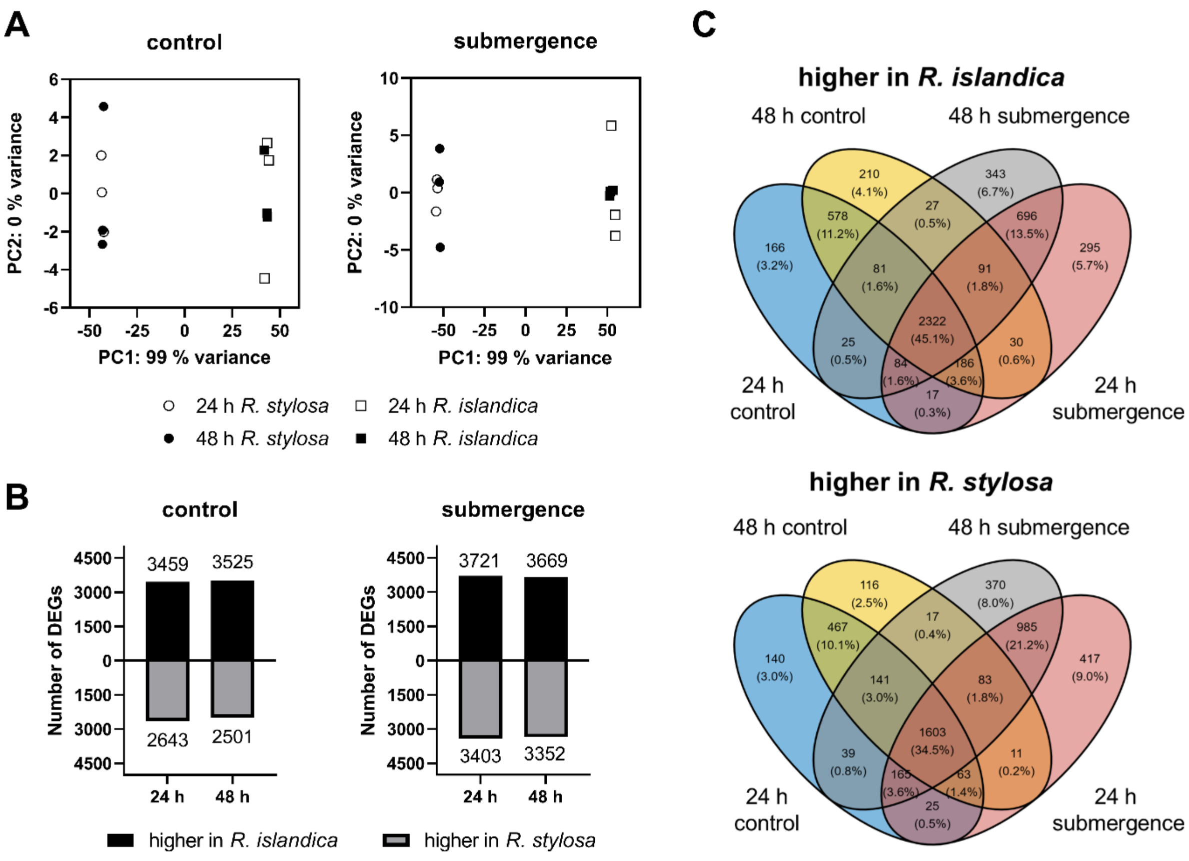

Supplementary Figure S7: Gene expression differences between *R. stylosa* and *R. islandica*. Plants were treated with submergence in day-night rhythm for 24 and 48 hours, and subsequently, young leaves were harvested for RNA extraction and RNA sequencing. (A) Principal component analysis of transcript levels of *R. stylosa* and *R. islandica* under control and submergence. (B) Number of differentially expressed genes (DEGs, Padj < 0.05 and |log2FC| > 2) between *R. stylosa* and *R. islandica*. (C) Overlap of species-specific DEGs between the two treatments and the two time points.

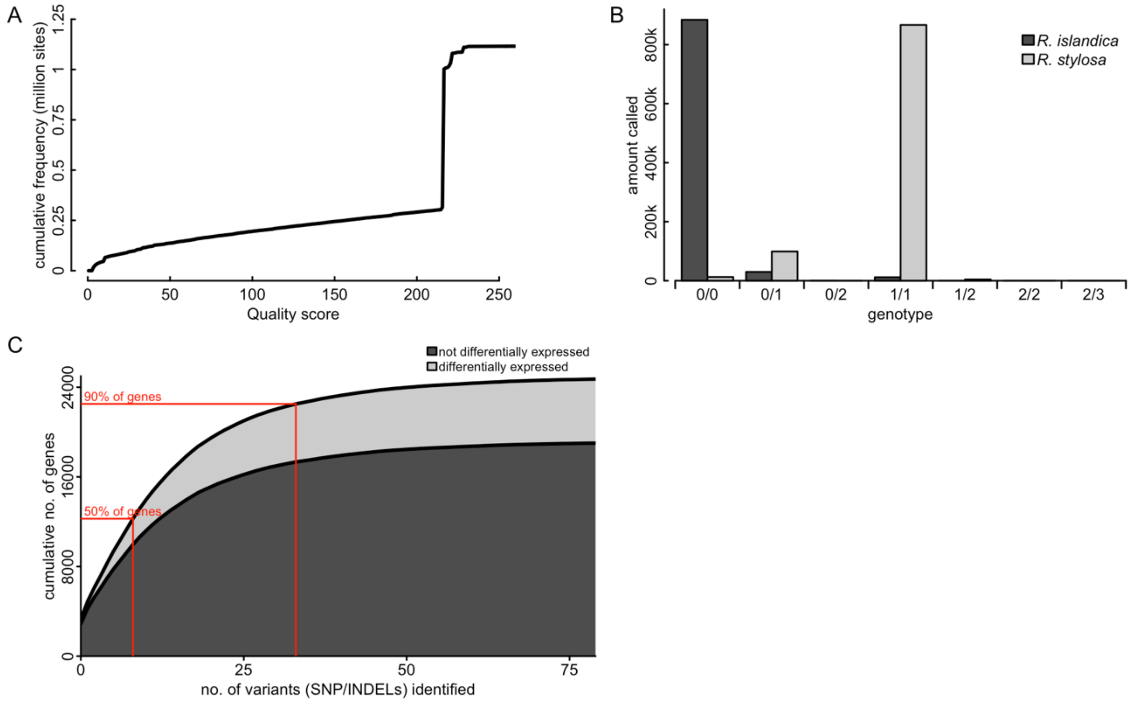

Supplementary Figure S8: Sequence variation between *R. islandica* and *R. stylosa*. (A) Cumulative frequency distribution of the Phred-scaled quality score for the assertion made on sequence variation that is an alternative to the *R. islandica* reference. (B) Counts of sequence variants compared to the *R. islandica* reference after removing low quality calls (<50) and removal of identical genotypic calls between *R. islandica* and *R. stylosa*. Here 0 refers to the reference sequence, whereas 1, 2 and 3 indicate alternative sequence variants (SNPs/INDELs) for that position. E.g. 0/1 indicates heterozygous with a reference and an alternative variant. (C) 62% of the observed variants were silent, in that they did not result in a predicted amino acid substitution. The remaining 38% of variants resulted in an altered protein. Genes with a sequencing depth >20 but without calls were considered mutation free. The resulting cumulative mutation load found in a gene is shown and their frequency split among differentially expressed (|log2FC| > 1 & Padj < 0.001) and non-regulated genes. The majority of genes have only a few amino acid substitutions, with 50% of the genes with less than 9 variants and 90 % less than 34 codon-altering mutations).

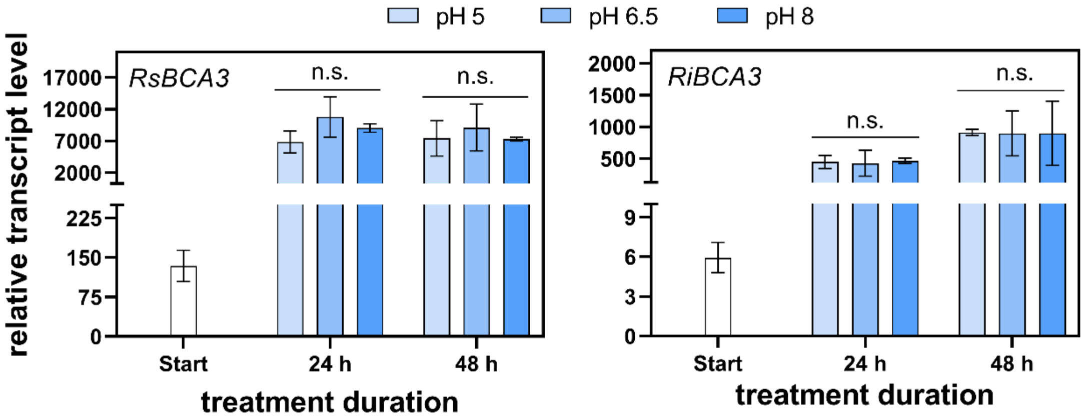

Supplementary Figure S9: *BCA3* induction is not dependent on pH of submergence water. RT-qPCR of *RsBCA3* and *RiBCA3* after different durations of submergence in light and darkness, with different pH of the flood water. Data are means +/- SD from three independent replicates. Data was normalized to *TUB6* and *ACT2*. n.s., no significant differences were observed between treatment types (two-way ANOVA, Tukey's HSD Test, $P < 0.05$).

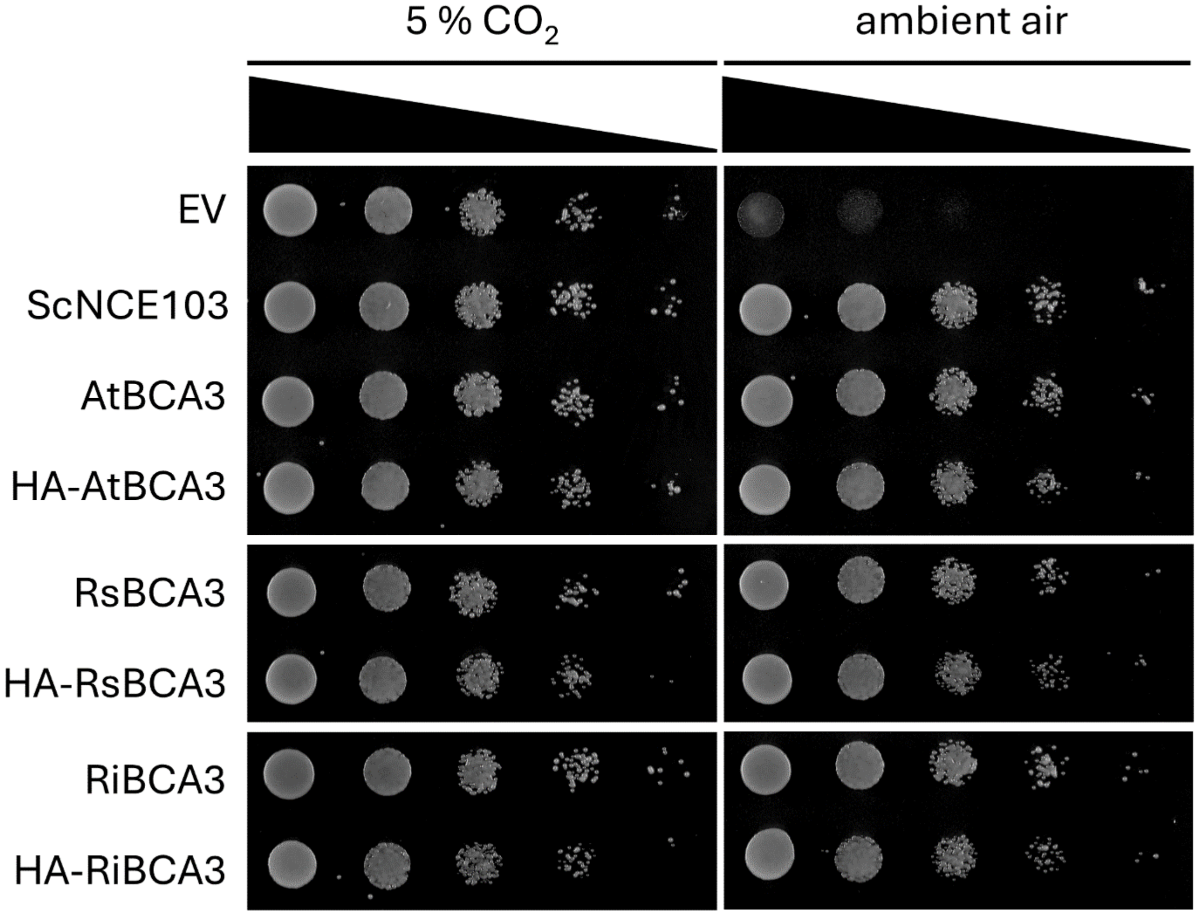

Supplementary Figure S10: *BCA3* from all three species codes for enzymatically active Carbonic Anhydrases. Yeast cells (ΔNCE103) were complemented with plasmids bearing the wildtype and HA-tagged coding sequences of *BCA3* against NCE103 as a positive and an empty vector (EV) control. Droplets are diluted 10^0 to 10^{-4} and grown under ambient air or 5 % CO_2. One representative of three independently conduced replicates is shown.

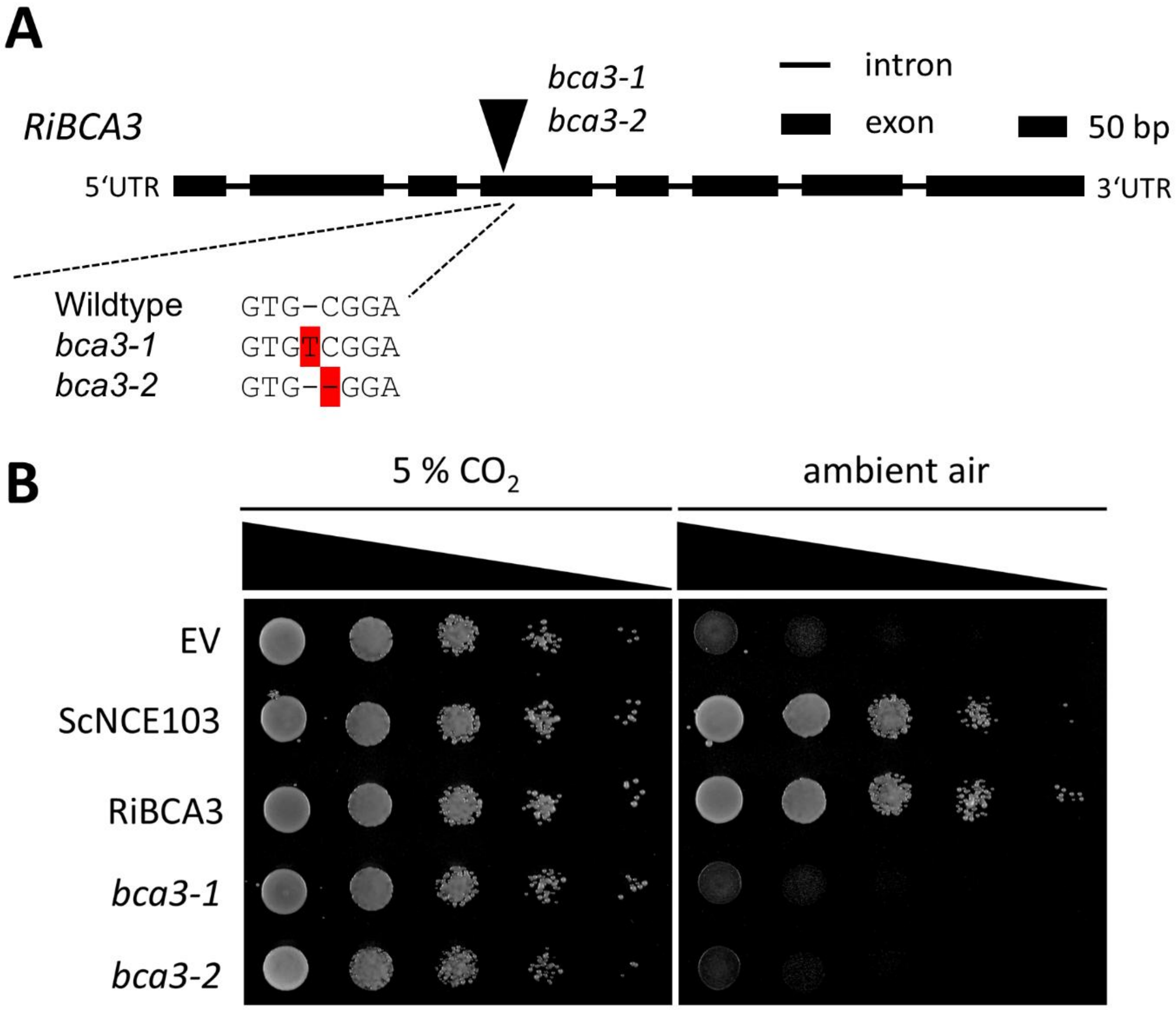

Supplementary Figure S11: Creation of *bca3* CRISPR-based knockout plants. (A) Gene model for *RiBCA3*. The arrow indicates the place of point mutations. Mutated bases are marked with red highlighting. (B) Introduced mutations disrupt BCA3 enzyme activity. Yeast cells (ΔNCE103) were complemented with plasmids bearing the wildtype and mutated coding sequences of *BCA3* against NCE103 as a positive and an empty vector (EV) control. Droplets are diluted 10^0 to 10^{-4} and grown under ambient air or 5 % CO_2. One representative of three independently conduced replicates is shown.

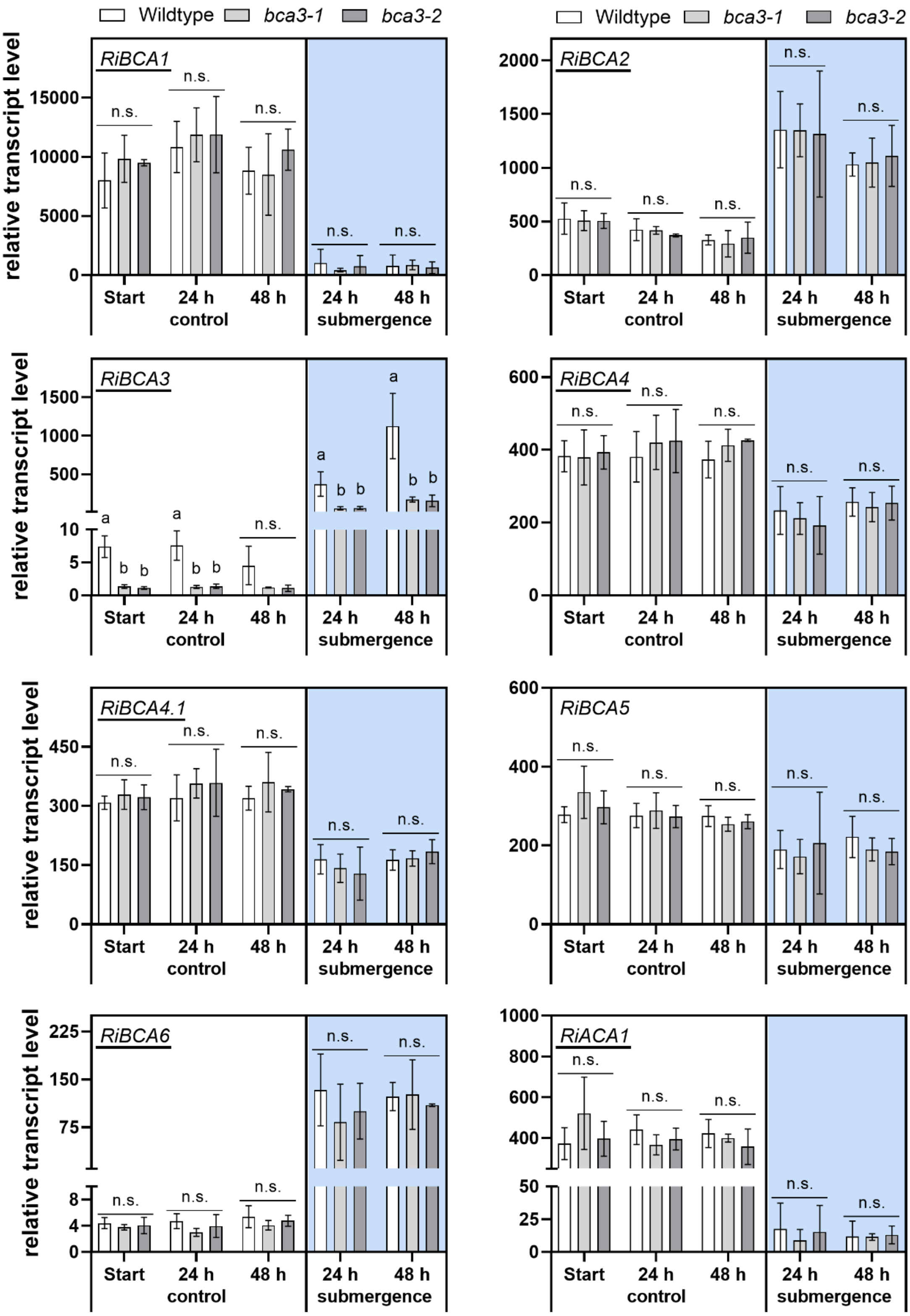

Supplementary Figure S12: Transcript levels for beta Carbonic anhydrases and alpha Carbonic anhydrase 1 (*ACA1*) in *R. islandica bca3* knockout plants. RT-qPCR of *RiBCA* genes and *RiACA1* after different durations of submergence. Data was normalized to *TUB6* and *ACT2*. Data are means +/- SD from three independent replicates. Different letters indicate significant differences between

genotypes; n.s., no significant differences between genotypes (one-way ANOVA for each time point, Tukey's HSD Test, $P < 0.05$). Significant differences between treatments in wildtype samples were marked with underlining gene names (one-way ANOVA, Tukey's HSD Test, $P < 0.05$).

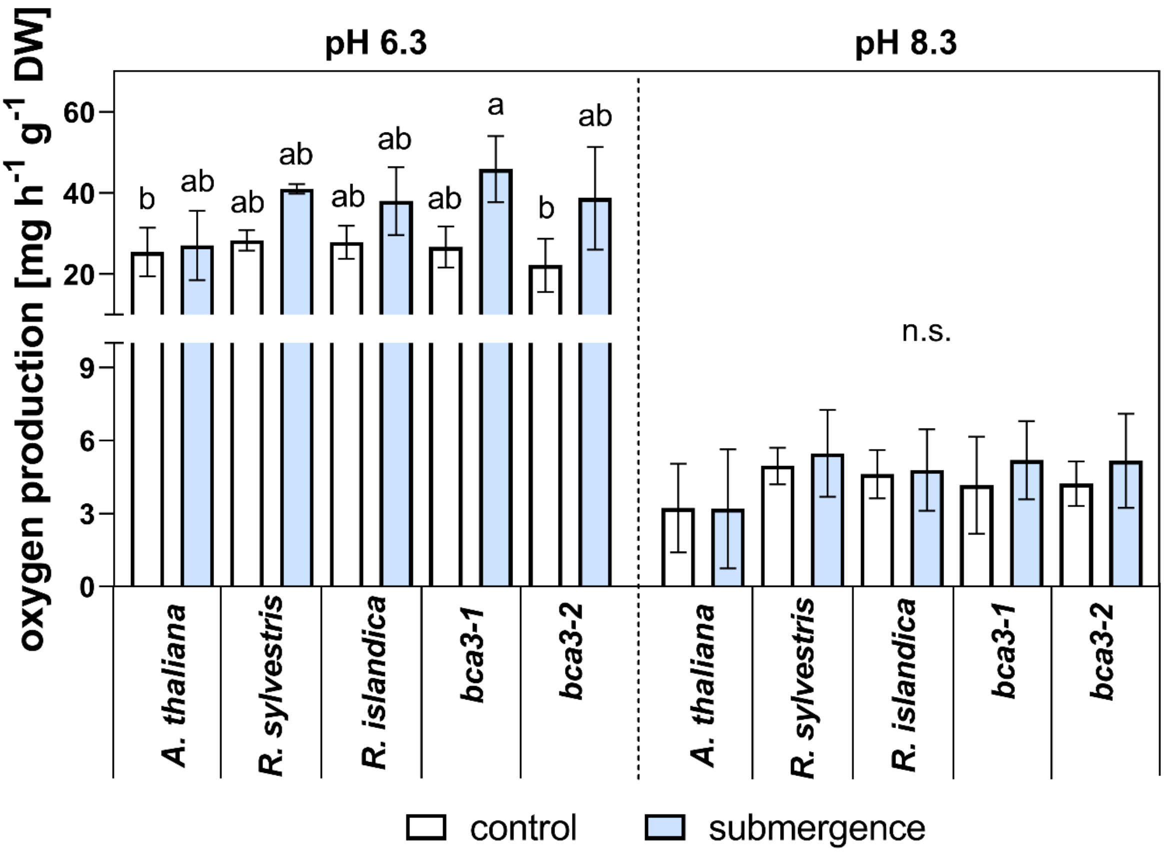

Supplementary Figure S13: Underwater photosynthesis of Arabidopsis, *R. sylvestris*, and *R. islandica* as well as *bca3* knockout lines. Oxygen production of leaves from control and submergence pretreated plants. Data are means +/- SD from three independent replicates. Different letters indicate significant differences within respective pH; n.s., no significant differences within respective pH (one-way ANOVA for each pH, Tukey's HSD Test, $P < 0.05$).

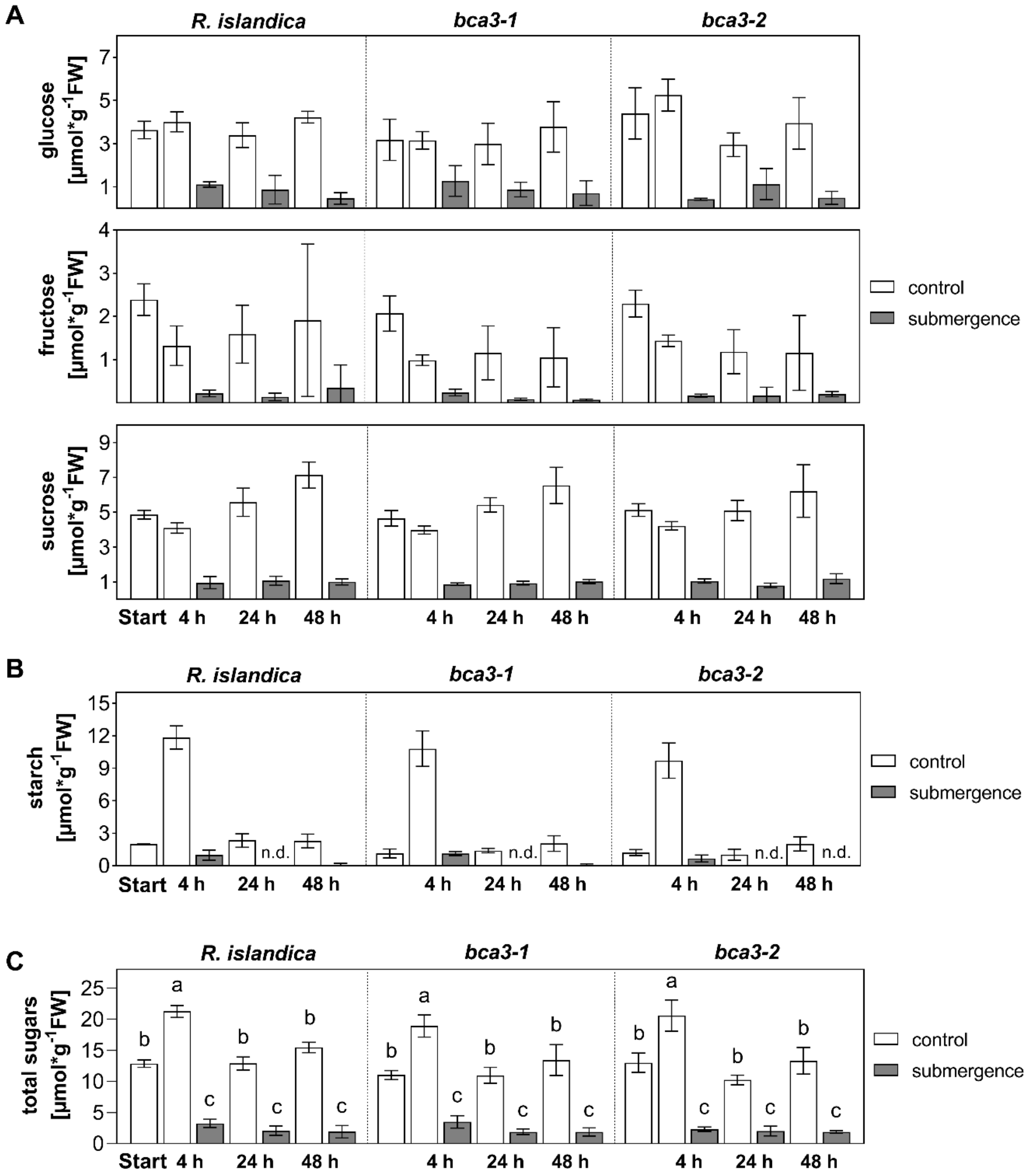

Supplementary Figure S14: Carbohydrate content in *R. islandica bca3* knockout plants after different durations of submergence. Soluble sugars (A, glucose, fructose, sucrose) and starch (B) were extracted from leaves. Data are means +/- SD from three independent replicates. (C) Sum of (A) and (B). Different letters indicate significant differences (two-way ANOVA, Tukey's HSD Test, P < 0.05). n.d., not detectable.

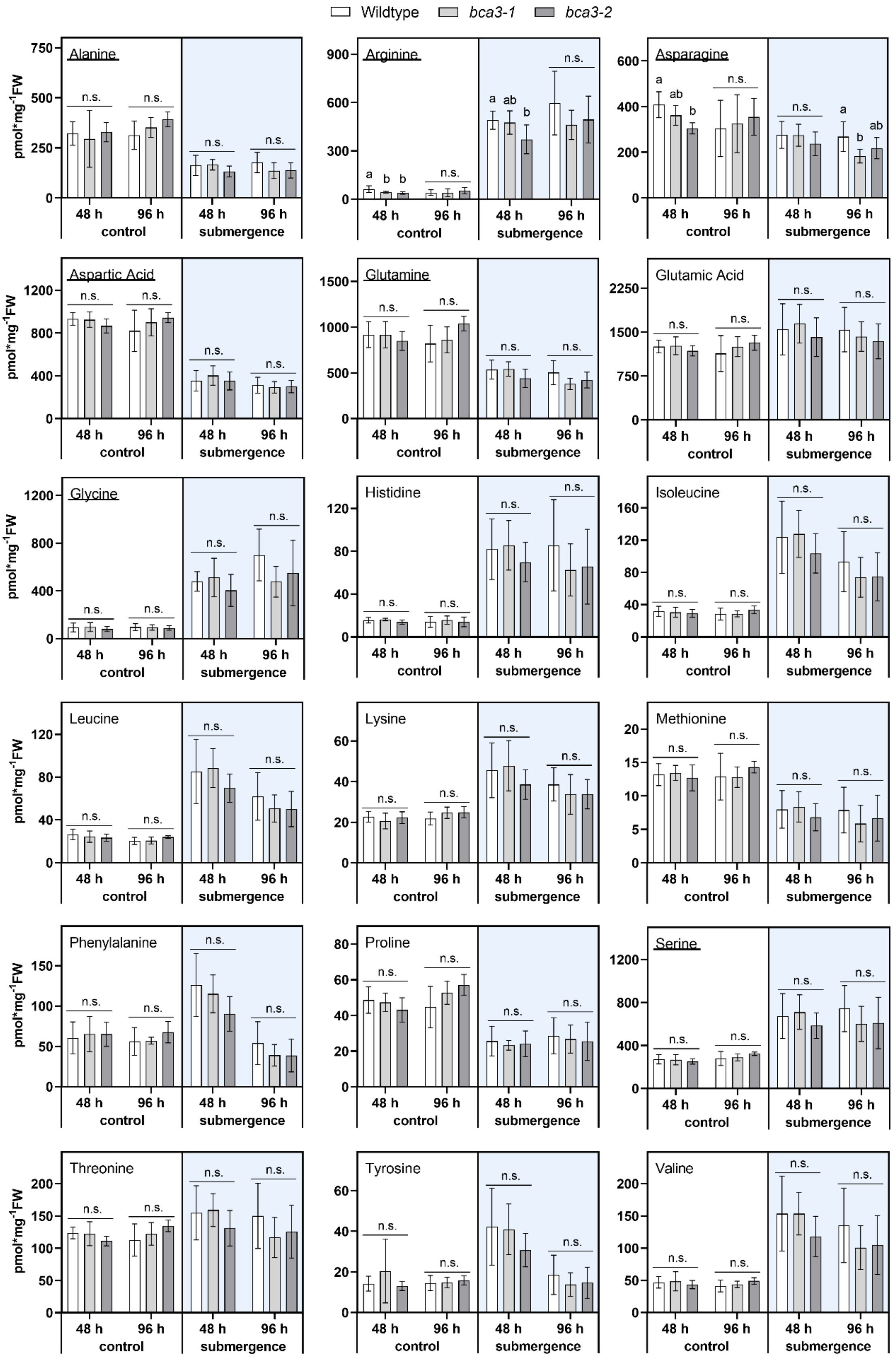

Supplementary Figure S15: Amino acid content in leaves of *R. islandica bca3* knockout plants after different durations of submergence. Data are means +/- SD from 6 samples out of three independent

experiments. Different letters indicate significant differences between genotypes; n.s., no significant differences between genotypes (one-way ANOVA for each time point, Tukey's HSD Test, $P < 0.05$). Amino acids with significant differences between treatments in wildtype samples were marked with underlining (one-way ANOVA, Tukey's HSD Test, $P < 0.05$).

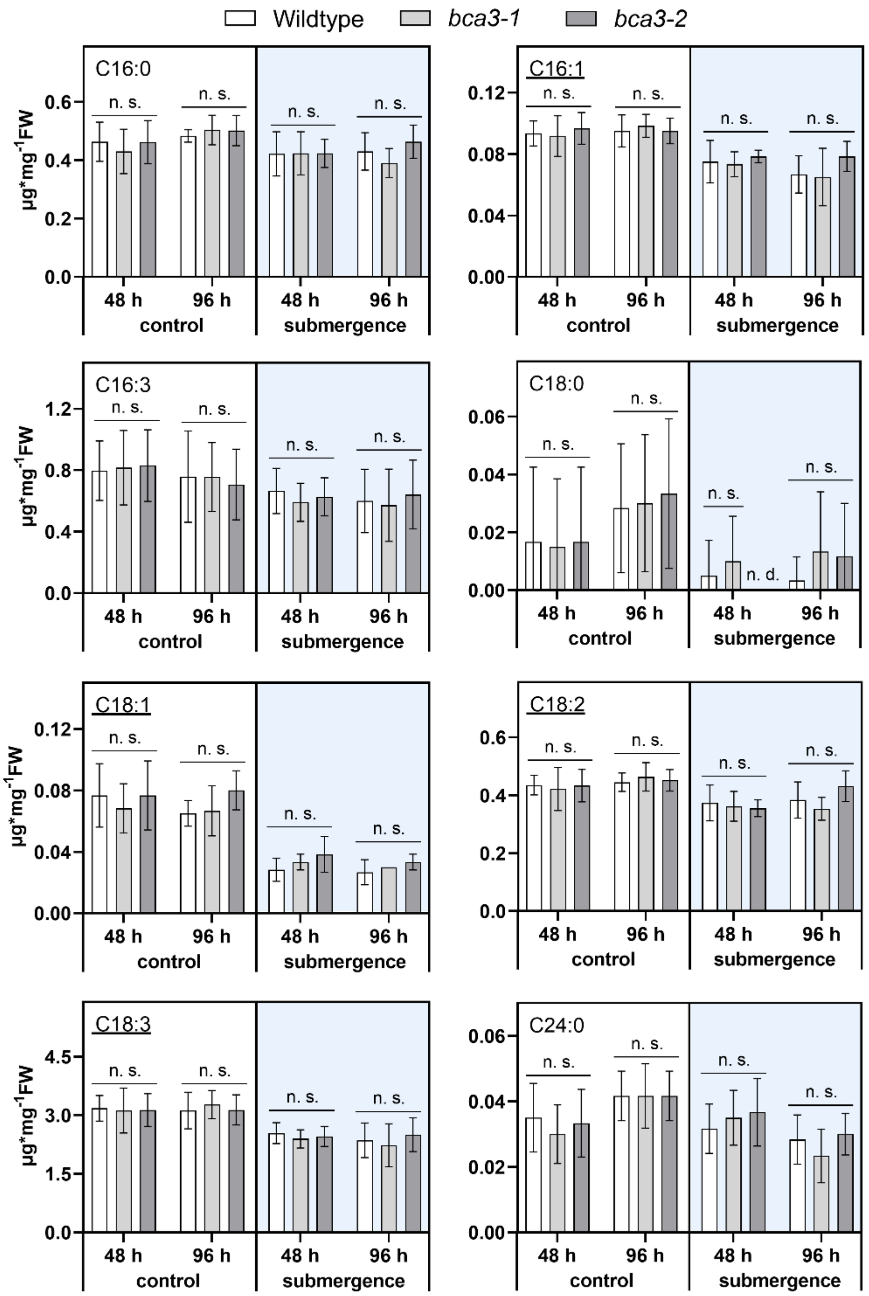

Supplementary Figure S16: Fatty acid content in leaves of *R. islandica bca3* knockout plants after different durations of submergence. Data are means +/- SD from 6 samples out of three independent experiments. n.s., no significant differences between genotypes (one-way ANOVA for each time point, Tukey's HSD Test, $P < 0.05$). Fatty acids with significant differences between treatments in wildtype samples were marked with underlining (one-way ANOVA, Tukey's HSD Test, $P < 0.05$).

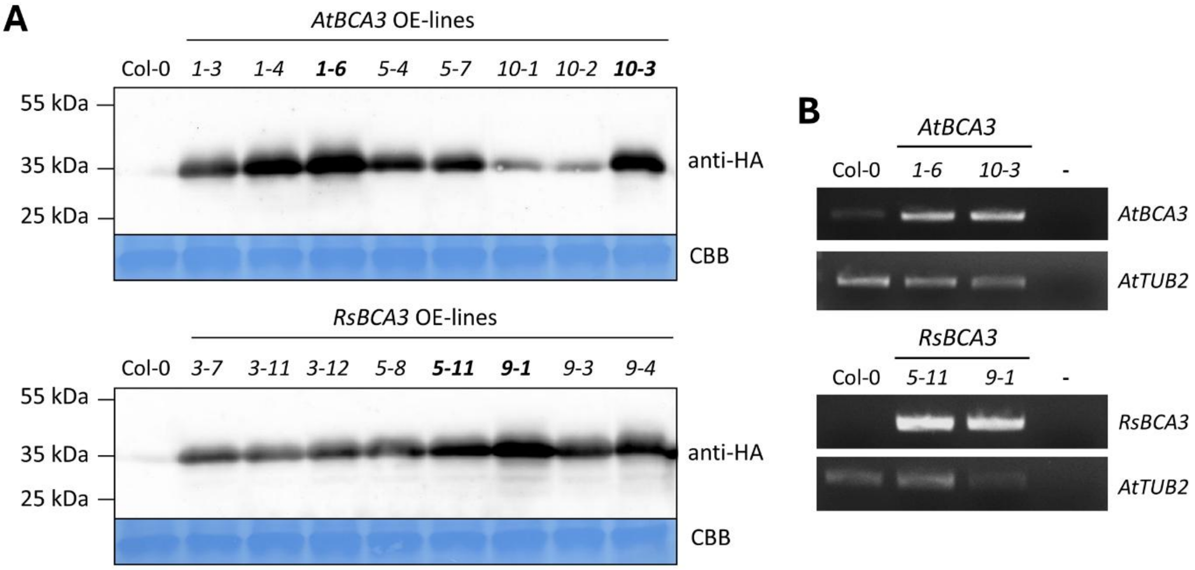

Supplementary Figure S17: Creation of Arabidopsis lines with *BCA3* overexpression. (A) Western blot of HA-tagged BCA3 in overexpression lines for *AtBCA3* and *RiBCA3*. Bold font, lines selected for further analysis. CBB, Coomassie brilliant blue loading control. (B) RT-PCR of *AtBCA3* and *RiBCA3* against *AtTUB2* in Col-0 and selected *BCA3* overexpression lines.

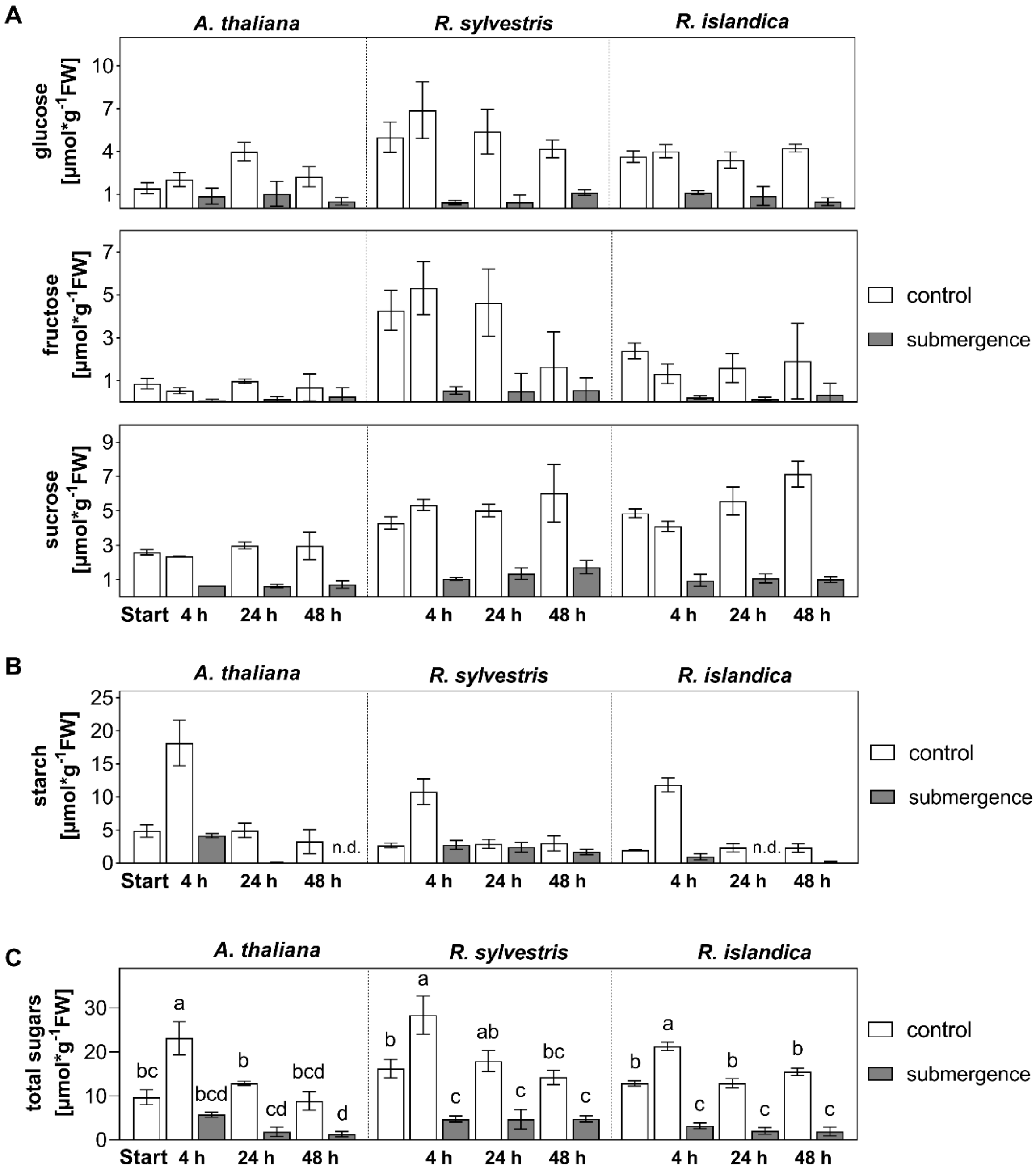

Supplementary Figure S18: Is the sugar content associated with submergence tolerance? Carbohydrate content in Arabidopsis and *Rorippa* plants after different durations of submergence in day-night rhythm. Soluble sugars (A, glucose, fructose, sucrose) and starch (B) were extracted from leaves. Data are means +/- SD from three independent replicates. (C) Sum of (A) and (B). Different letters indicate significant differences (two-way ANOVA, Tukey's HSD Test, $P < 0.05$). n.d., not detectable.

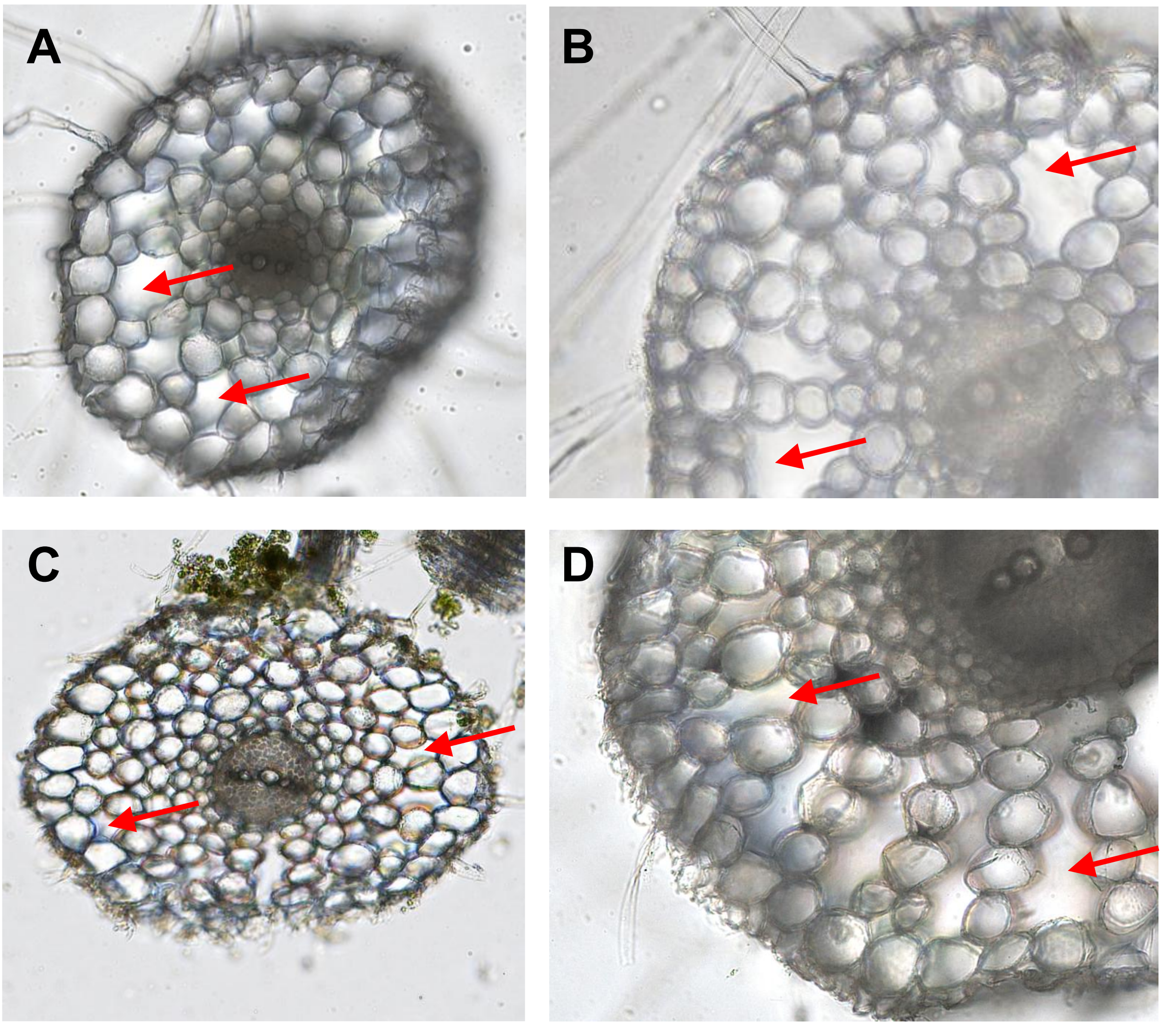

Supplementary Figure S19: Aerenchyma formation in root cross sections of *R. islandica* and *R. sylvestris*. (A, B) *R. islandica*; (C, D) *R. sylvestris*. Red arrows point at schizogenous aerenchyma.

Supplementary Tables

Supplementary Table S1: Mapping statistics. Percentage of RNA sequencing reads that mapped to the reference genome of *R. islandica*, and percentage of uniquely mapped reads. Mapping was performed with kallisto.

Sample	Reads	pseudoaligned [%]	uniquely pseudoaligned [%]
R. islandica 24 h control I	46,442,782	88.7	83.8
R. islandica 24 h control II	76,202,305	87.5	82.8
R. islandica 24 h control II	41,548,953	87.7	82.9
R. islandica 48 h control I	31,074,683	89.8	84.8
R. islandica 48 h control II	44,552,615	88	83.1
R. islandica 48 h control II	37,327,968	88.5	83.6
R. islandica 24 h submergence I	41,063,340	88.8	83.4
R. islandica 24 h submergence II	49,901,815	89.1	83.6
R. islandica 24 h submergence III	41,147,939	89	83.6
R. islandica 48 h submergence I	33,342,986	80.3	75.3
R. islandica 48 h submergence II	38,506,569	88	82.4
R. islandica 48 h submergence III	42,803,260	87.5	82

Sample	Reads	pseudoaligned [%]	uniquely pseudoaligned [%]
R. stylosa 24 h control I	43,841,378	81.3	76.1
R. stylosa 24 h control II	34,507,010	81.3	76.3
R. stylosa 24 h control II	44,835,981	81.8	76.7
R. stylosa 48 h control I	46,861,635	79.2	74.4
R. stylosa 48 h control II	45,501,284	81.6	76.6
R. stylosa 48 h control II	47,759,293	81.8	76.7
R. stylosa 24 h submergence I	29,982,032	80.5	75.3
R. stylosa 24 h submergence II	44,690,206	80.5	75.2
R. stylosa 24 h submergence III	32,067,114	79.6	74.4
R. stylosa 48 h submergence I	34,464,159	79.6	74.1
R. stylosa 48 h submergence II	31,178,841	79.1	73.8
R. stylosa 48 h submergence III	38,423,252	78.8	73.6

Supplementary Table S2: Primers used in this study.

Primer	Sequence
RsBCA3_fw	GCACTAGCCAAGAGCCAGAG
RsBCA3_rev	AGTCGAGGTCCCAGAGATCA
AtTUB2_fw	CTCAAGAGGTTCTCAGCAGTA
AtTUB2_rev	TCACCTTCTTCATCCGCAGTT
AtPP2A_qPCR_fw	TAACGTGGCCAAAATGATGC
AtPP2A_qPCR_rev	GTTCTCCACAACCGCTTGGT
AtUBC9_qPCR_fw	TCACAATTTCCAAGGTGCTGC
AtUBC9_qPCR_rev	TCATCTGGGTTTGGATCCGT
RsRiACT2_qPCR_fw	CACTGTGCCAATCTACGAGG
RsRiACT2_qPCR_rev	TACAATTTCCCGCTCTGCTG
RsTUB6_qPCR_fw	CACGGCAATGTTCAGGAGAA
RsTUB6_qPCR_rev	ACAAGGTCGTTCATGTTGCT
RiTUB6_qPCR_fw	AACAGATGTCGTAAAGGGCC
RiTUB6_qPCR_rev	TGTCTTCCCATCACCAAAGG
RstylTUB6_qPCR_fw	GGGCATTACACTGAAGGAGC
RstylTUB6_qPCR_rev	AGAGTTCCCATTCCAGACCC
RiBCA1_qPCR_fw	TCGTTCTTCTTCCACCGTCG
RiBCA1_qPCR_rev	GGGCTCGTTACGGATAAGGG
RiBCA2_qPCR_fw	CGGAGCTTCAGACAGTCTCG
RiBCA2_qPCR_rev	TTTGGGCTTTGACCTTTGGC
AtBCA3_qPCR_fw	CACTCTAATGTTGGTGCGGC
AtBCA3_qPCR_rev	AACTCGGTCTTAGTGGGAGC
RsBCA3_qPCR_fw	GGCCAAGAACAGGATCAAGC
RsBCA3_qPCR_rev	TCACCGCTTCCTTCTCACAG
RiBCA3_qPCR_fw	GATGCAGCTCCCACTAAGAC
RiBCA3_qPCR_rev	GTTGCACTGATCGTCAAAGC
RstylBCA3_qPCR_fw	ACAACTGTGAGAAGGAAGCG
RstylBCA3_qPCR_rev	CTCCTCTGATGGTGAGCTTG
RiBCA4_qPCR_fw	GCTGTTGTGGTGGGATTAAGG
RiBCA4_qPCR_rev	TGATGCGCCTATCTTCACCC
RiBCA4.1_qPCR_fw	TGTTTCATGTTCTGCTGCGC
RiBCA4.1_qPCR_rev	TGATCTTCTCTGCAGCGACG
RiBCA6_qPCR_fw	CGTCGCCTAGTCTCTGCAAC
RiBCA6_qPCR_rev	AACCATGTTCGTCCTCCTGG
RsARL_qPCR_fw	TGTGCTTGTTGGTCTCACTG
RsARL_qPCR_rev	AAGCAGCATAAACGGAGGAG
RiARL_qPCR_fw	CGTCTCAGAACAGTCCAAGG
RiARL_qPCR_rev	AGCATAAAGGGAGGAGGAGG
RsADH1_qPCR_fw	TGGTTTGTCATGCTCTCTCG
RsADH1_qPCR_rev	GAAGGTGCTAGAATCGCTGG
RiADH1_qPCR_fw	GAAGGTGCTAGAATCGCTGG

RiADH1_qPCR_rev	GCTGAACTGGTTTGTCATGC
RsLBD41_qPCR_fw	TCTCGCAAAGTTCTATGGCC
RsLBD41_qPCR_rev	CATAACAAACCCACCGAGCC
RiLBD41_qPCR_fw2	TCTATGGTTCGGTGGGTTTG
RiLBD41_qPCR_rev2	TCGGTTCACCTTTCAACACC
RiBCA3_CRISPR_guide_fw	ATTGGAAACACGTGAATCCGCAC
RiBCA3_CRISPR_guide_rev	AAACGTGCGGATTCACGTGTTTC
attB1_fw	GGGGACAAGTTTGTACAAAAAAGCAGGCT
attB2_rev	GGGGACCACTTTGTACAAGAAAGCTGGGT
nHA_pDD506_fw	TGACATCGATGGCATACCCATACGACGT
AtBCA3_pDD506_fw	GTCCATCGATGTCGACAGAGTCGTACGAA
AtBCA3_pDD506_rev	AATCTCGAGTTAAGACAAGGCAAAGGCAGG
RsBCA3_pDD506_fw	GCAATCGATGTCGACGGAGTCGTATGAAG
RiRsBCA3_pDD506_rev	ATTCTCGAGTTAAGACAAGGCAAAAGCAGGG
RiBCA3_pDD506_fw	GCGATCGATGTCGACAGAGTCGTATGAAGAT
ScNCE103_pDD506_fw	TAATCGATGAGCGCTACCGAATCTTC
ScNCE103_pDD506_rev	ATACTCGAGCTATTTTGGGGTAACTTTTGTGT
AtBCA3_attB1_fw	AAAAAGCAGGCTTAATGTCGACAGAGTCGTACGAA
AtBCA3_attB2_rev	AGAAAGCTGGGTTTTAAGACAAGGCAAAGGCAGG
RsBCA3_attB1_fw	AAAAAGCAGGCTTAATGTCGACGGAGTCGTACGAA
RsBCA3_attB2_fw	AGAAAGCTGGGTTTTAAGACAAGGCAAAAGCAGG
RiBCA3_attB1_fw	AAAAAGCAGGCTTAATGTCGACAGAGTCGTA
RiBCA3_attB2_rev	AGAAAGCTGGGTATTAAGACAAGGCAAAAGC

Supplementary Datasets *(as separate files)*

Supplementary Dataset S1: Transcript levels and DEGs (Padj < 0.05 and |log2FC| > 2) for *R. islandica* and *R. stylosa* after 24 and 48 h submergence compared with control conditions. DEG analysis was performed with DESeq2. GO enrichment analysis from DEGs was performed with PANTHER.

Supplementary Dataset S2: Comparison of this dataset to the previous publication (van Veen et al. 2024). Transcript levels were quantified after assignment to previously defined orthogroups (OGs) and compared among the seven species. Commonly regulated OGs (cutoff 12 out of 7 species x 2 time points) were subjected to GO enrichment analysis with PANTHER.

Supplementary Dataset S3: Transcript levels and DEGs (Padj < 0.05 and |log2FC| > 2) between the two species *R. islandica* and *R. stylosa* under submergence and control conditions. DEG analysis was performed with DESeq2. GO enrichment analysis from DEGs was performed with PANTHER.

Supplementary Dataset S4: Selection of candidate genes that could be associated with higher submergence tolerance in *R. islandica* compared with *R. stylosa*. Genes were selected as submergence-induced DEGs in *R. islandica* in the comparison submergence vs control (Dataset S1), and as higher expressed in *R. islandica* compared with *R. stylosa* (Dataset S3).

Supplementary Dataset S5: List of sequence variations between *R. islandica* and *R. stylosa*. GeneID, *R. islandica* gene identifier; POS, position in coding sequence; REF, nucleobase in reference sequence; ALTfound, all nucleobase variants observed in the dataset; ALT, respective nucleobase variation; depth, sequencing depth; ISL, occurrence of sequence variation in *R. islandica*; STY, occurrence of sequence variation in *R. stylosa*; variantID, ID of the sequence variation; ProteinID, *R. islandica* protein identifier; remaining columns, results of the BLAST search against *R. islandica* proteome.

Supplementary Dataset S6: Heatmap of photosynthesis-associated genes. Transcripts with homology to previously defined photosynthesis genes (MapMan categories, available at https://github.com/usadellab/usadellab.github.io/tree/master/MapManJS) were selected. Values are log2FC of submergence vs control for 24 and 48 h treatment in *R. stylosa* and *R. islandica*, as indicated by the scale. Transcripts with no significant changes (Padj > 0.05) are shown in grey. NA, no homologous gene found in the *R. islandica* genome, or no transcript detected.
